# Predicting Outcomes from Engagement With Specific Components of an Internet-Based Physical Activity Intervention With Financial Incentives: Process Analysis of a Cluster Randomized Controlled Trial

**DOI:** 10.2196/11394

**Published:** 2019-04-19

**Authors:** Jennifer M Murray, David P French, Christopher C Patterson, Frank Kee, Aisling Gough, Jianjun Tang, Ruth F Hunter

**Affiliations:** 1 Centre for Public Health School of Medicine, Dentistry and Biomedical Sciences Queen's University Belfast Belfast United Kingdom; 2 Manchester Centre of Health Psychology School of Health Sciences University of Manchester Manchester United Kingdom; 3 School of Agricultural Economics and Rural Development Renmin University of China Beijing China

**Keywords:** physical activity, workplace, randomized controlled trial, behavior, maintenance, motivation

## Abstract

**Background:**

Investigating participant engagement and nonusage attrition can help identify the likely *active ingredients* of electronic health interventions. Research on engagement can identify which intervention components predict health outcomes. Research on nonusage attrition is important to make recommendations for retaining participants in future studies.

**Objective:**

This study aimed to investigate engagement and nonusage attrition in the Physical Activity Loyalty (PAL) scheme, a 6-month complex physical activity intervention in workplaces in Northern Ireland. The intervention included financial incentives with reward redemption and self-regulation techniques. Specific objectives were (1) to determine whether engagement in specific intervention components predicted physical activity at 6 months, (2) to determine whether engagement in specific intervention components predicted targeted mediators at 6 months, and (3) to investigate predictors of nonusage attrition for participants recording daily activity via the PAL scheme physical activity monitoring system and logging onto the website.

**Methods:**

Physical activity was assessed at baseline and 6 months using pedometers (Yamax Digiwalker CW-701, Japan). Markers of engagement and website use, monitoring system use, and reward redemption were collected throughout the scheme. Random-effects generalized least-squares regressions determined whether engagement with specific intervention components predicted 6-month physical activity and mediators. Cox proportional hazards regressions were used to investigate predictors of nonusage attrition (days until first 2-week lapse).

**Results:**

A multivariable generalized least-squares regression model (n=230) showed that the frequency of hits on the website’s monitoring and feedback component (regression coefficient [*b*]=50.2; SE=24.5; *P*=.04) and the percentage of earned points redeemed for financial incentives (*b*=9.1; SE=3.3; *P*=.005) were positively related to 6-month pedometer steps per day. The frequency of hits on the discussion forum (*b*=−69.3; SE=26.6; *P*=.009) was negatively related to 6-month pedometer steps per day. Reward redemption was not related to levels of more internal forms of motivation. Multivariable Cox proportional hazards regression models identified several baseline predictors associated with nonusage attrition. These included identified regulation (hazard ratio [HR] 0.88, 95% CI 0.81-0.97), recovery self-efficacy (HR 0.88, 95% CI 0.80-0.98), and perceived workplace environment safety (HR 1.07, 95% CI 1.02-1.11) for using the physical activity monitoring system. The EuroQoL health index (HR 0.33, 95% CI 0.12-0.91), financial motivation (HR 0.93, 95% CI 0.87-0.99), and perceived availability of physical activity opportunities in the workplace environment (HR 0.96, 95% CI 0.93-0.99) were associated with website nonusage attrition.

**Conclusions:**

Our results provide evidence opposing one of the main hypotheses of self-determination theory by showing that financial rewards are not necessarily associated with decreases in more internal forms of motivation when offered as part of a complex multicomponent intervention. Identifying baseline predictors of nonusage attrition can help researchers to develop strategies to ensure maximum intervention adherence.

**Trial Registration:**

ISRCTN Registry ISRCTN17975376; http://www.isrctn.com/ISRCTN17975376 (Archived by WebCite at http://www.webcitation.org/76VGZsZug)

## Introduction

### Background

The worldwide *pandemic* of physical inactivity [1] requires innovative approaches to increasing population physical activity levels with a view to achieving long-term maintenance [2]. Physical activity interventions that can be delivered through less costly channels (eg, internet, telephone, or post) than those requiring direct contact, in the interest of reaching as many participants as possible, are needed [3].

An example of an internet-delivered intervention was the Physical Activity Loyalty (PAL) scheme, implemented in workplaces in Northern Ireland (NI). The PAL scheme was a complex physical activity intervention that offered financial incentives and other behavior change techniques delivered via the study website to increase workplace physical activity (recorded by outdoor sensors located within 2 km of the workplace). Paradoxically, results showed that there was a small but significant decline in pedometer steps per day at 6 months relative to the baseline for the intervention group compared with controls, which dissipated at 12 months [4]. Mediation and moderation analyses showed that decreases in physical activity were partially mitigated by positive indirect effects through the constructs of integrated regulation, intrinsic motivation, and habit measured at 6 months, whereas the negative intervention effect was moderated by participants’ perceptions of availability of physical activity opportunities in the workplace environment [5]. The analyses reported in this paper aimed to provide further insight to the mechanisms of behavior change for participants in the PAL scheme by examining usage rates for specific intervention components, predictors of usage rates, and whether usage was related to study outcomes.

The concept of *engagement* may be defined in terms of the level of exposure to and use of an intervention and the amount of skills practice involved (ie, completing activities or exercises to acquire knowledge or learn behavior relevant to the target outcome) [6]. A participant’s level of engagement determines the extent to which they receive the intended intervention, and research on engagement is useful for identifying which intervention components are associated with health outcomes [7,8]. Investigating engagement in the different components of the intervention separately may help uncover which aspects of the intervention were beneficial (or detrimental) for increasing physical activity behavior. Thomson et al, for example, examined the intervention engagement indicators (both singly and combined) in relation to several health behaviors in a broad lifestyle intervention and recommended the use of single engagement indicators, relevant to each intervention component, for predicting health outcomes [8]. Examining engagement in this way may help identify the key *active ingredients* [9,10] for refinement in future studies. Other authors have noted that although previous studies have focused on the comparative effectiveness of Web-based interventions, they have neglected to test hypotheses about the mechanisms of action [11]. Understanding *how* and *why* interventions affect outcomes will enable the development of more efficient Web-based interventions [11]. This paper contributes to filling a gap in the research base by investigating the relationship between intervention engagement and mediator outcomes targeted by the PAL intervention. Thus, it reflects guidance provided by the Medical Research Council on conducting process evaluations, which promotes the understanding of *cause* as a key feature [12].

An issue observed to impact Web-based interventions is the tendency for a substantial proportion of participants to discontinue use of the intervention before the intervention ends [13]. Nonusage attrition refers to the phenomenon of participants ceasing intervention use before the end of the intervention period, which seems to particularly affect Web-based interventions [13]. For example, previous Web-based physical activity interventions targeting healthy, sedentary adults define nonusage attrition as occurring when the participant has a 2- week lapse from using the intervention [14,15]. To determine how successful Web-based interventions are for achieving health behavior change, it is important to understand participants’ nonusage patterns and their influencing factors. Thus, we might be able to make recommendations for participant retention in future intervention studies. This is important given that lack of participant engagement and high levels of nonusage attrition are factors that can impede researchers’ ability to appropriately test hypotheses in intervention studies [8,16].

### Objectives

The objectives of this paper were (1) to determine whether levels of engagement in different components of the intervention predicted physical activity measured 6 months post baseline for participants assigned to the intervention group, (2) to determine whether levels of engagement in different components of the intervention predicted psychosocial variables (ie, mediators) targeted by the intervention at 6 months post baseline, and (3) to investigate rates of nonusage attrition for participants recording daily activity via the PAL scheme physical activity monitoring system and logging onto the PAL scheme website and baseline predictors of nonusage attrition (ie, sociodemographic, mediator, environmental, and physical activity variables) for participants in the intervention group.

## Methods

### Overview

The PAL scheme was a cluster randomized controlled trial of a complex, 6-month multicomponent workplace intervention targeting inactive employees in workplaces in Belfast and Lisburn city centers in NI to increase their physical activity during working hours [17]. The underpinning theoretical framework was based on the learning theory [18], self-regulation control theory [19], social cognitive theory [20], and self-determination theory [21]. The scheme included a novel physical activity tracking system (with sensors in outdoor locations within 2 km of the workplace) and Web-based monitoring (ie, self-monitoring, prompts and cues, habit formation, and adding objects to the environment). The main intervention component was the provision of financial incentives [22] with *points* accumulated depending on participants’ minutes of walking (ie, 1 *point* for 1 min of physical activity with a notional monetary value of £0.03 for a maximum of 30 min per day) and could be redeemed for rewards at local businesses. Maps of walking routes and examples of physical activity opportunities were provided on the website (ie, instruction on how to perform the behavior). Sensors were operational during working hours (ie, 7 am-7 pm, Monday-Friday). Other behavior change techniques included regular tailored motivational emails (ie, prompts), tailored feedback, and links to other resources (eg, physical activity and healthy eating advice) [23]. Discussion forums on the website provided a platform for participants to contact researchers and other participants (ie, social support). Participants randomly assigned (in clusters) to the control arm received no intervention during the 6-month intervention period but were placed on a waiting list to participate in the scheme at the end of the study period (ie, 12 months). A more detailed overview of the trial procedures, including the Consolidated Standards of Reporting Trials (CONSORT) flow diagram, and intervention program has been published [4] and is summarized in Multimedia Appendix 1. The Consolidated Standards of Reporting Trials of Electronic and Mobile HEalth Applications and onLine TeleHealth (CONSORT-EHEALTH) checklist has been completed for this study [24].

### Data Collection

Outcome data were collected at baseline (sociodemographic, mediator, environmental, and physical activity variables), 6 months (mediator and physical activity variables), and 12 months (physical activity only). Data on daily physical activity captured via the PAL scheme physical activity monitoring system (ie, dates and minutes), website usage (ie, dates, number of hits, and minutes), and reward redemption (ie, number of earned points and proportion redeemed) were collected throughout the 6-month intervention period.

### Outcome Measurements

#### Engagement Variables

A total of 3 markers of overall intervention engagement (ie, daily physical activity captured via the PAL scheme physical activity monitoring system, use of the PAL website, and reward redemption) were tracked throughout the 6-month intervention period and the following variables were derived:

1. *Percentage of intervention days during which participants walked for at least 10 min captured via the PAL scheme physical activity monitoring system over the 6-month intervention period.* This captured participants’ engagement with the physical activity monitoring system component of the intervention (ie, their willingness to practice physical activity behavior in the workplace and earn points to incentivize their physical activity). Government recommendations suggest that adults (aged 18-65 years) should accumulate 150 mins per week of moderate-intensity physical activity or 75 mins per week of vigorous-intensity physical activity, or an equivalent combination of both, in blocks of at least 10-min duration [25]. Recommendations from the Chief Medical Office emphasize the importance of daily physical activity and suggest the accumulation of 30 min of at least moderate-intensity physical activity on most, preferably all, days of the week [26]. Therefore, engagement was measured in terms of days, and only days with at least 10 min of recorded activity were counted.
2. *Percentage of intervention weeks during which participants logged onto the PAL website at least once over the 6-month intervention period.* Although there was no specific guidance for *intended* *use* of the PAL website (ie, there was no recommendation for how often participants should log on), research shows that the typical Web-based intervention is meant to be used once a week [27], and previous studies have categorized a log-in frequency of once per week as being high [28]. Therefore, engagement was measured in terms of weeks, and only weeks during which participants logged in at least once were counted.
3. *Percentage of earned points redeemed over the 6-month intervention period.* Aside from earning points by recording activity via the physical activity monitoring system, this indicator captured whether participants were interested in redeeming their earned points for financial rewards to incentivize their physical activity behavior.

Engagement with the different aspects of the PAL website was assessed as the *frequency of hits on each intervention component* *for every 10 days the participant accessed the website* *and the* *t* *otal number of intervention components accessed on the website at least once (range 0-6).* Research shows that measures of time spent on the study website may not accurately capture engagement with the intervention. For example, although Web-based interventions with unstructured access facilitate tailoring and flexibility, they enable users to multitask by opening multiple Web pages or undertaking other activities, complicating the measurement of intervention engagement [29]. As participants in the PAL scheme had complete freedom to choose how they used the website, a higher frequency of hits on a particular website component across the days on which the participant chose to log on was expected to reflect a higher level of interest (and willingness to engage) in that aspect of the intervention. This was also in line with previous studies of engagement in Web-based interventions using log-in frequency as a measure of engagement [28,30-34].

The 6 intervention components participants could access on the website were as follows:

1. *Monitoring and feedback:* Data and visual representation (ie, graphics) of the participant’s activity over the intervention period for self-monitoring purposes (ie, self-monitoring and feedback and goal setting)
2. *Rewards:* Platform for participants to view their earned and bonus points, information on available rewards, and how to redeem points (ie, immediate reward contingent on behavior change)
3. *Maps:* Maps of sensor locations and example walking routes for planning of physical activity (ie, information on when and where to perform physical activity and action planning)
4. *Health information (physical activity):* Physical activity facts and information, health benefits, safety tips, and tips for a physically active lifestyle (ie, provision of information about health benefits of physical activity)
5. *Health information (other):* Information related to healthy eating, smoking, alcohol consumption, and stress reduction (ie, provision of information about health benefits of other health behaviors)
6. *Discussion forums:* Platform for participants to contact researchers and other participants to ask questions, make enquiries, raise concerns, and respond to comments (ie, social support).

#### Nonusage Attrition: Recording Activity Via the Physical Activity Monitoring System and Website Use

Nonusage attrition was considered to occur if a participant had at least a 2-week lapse from use [14,15]. Nonusage attrition for recording activity via the physical activity monitoring system was measured as the number of days until the first 2-week lapse from recording activity. Website nonusage attrition was measured as the number of days until the first 2-week lapse from logging onto the website.

#### Physical Activity

The primary outcome was steps per day objectively measured over 7 days using sealed pedometers (Yamax Digiwalker CW-701, Japan) [35-37] and considered valid if the participant provided more than or equal to 250 steps per day for 3 or more days. This was collected at baseline, 6 months, and 12 months. The primary outcome assessment was distinct from the data collected from the PAL physical activity monitoring system. Specifically, the physical activity monitoring system was used to capture data on the minutes of workplace physical activity undertaken in the outdoor workplace environment by intervention group participants within the core hours of 7 am to 7 pm, Monday to Friday. These data were used to compute participants’ points, were redeemable for financial incentives, and were available on the study website as a self-monitoring tool. The primary outcome assessment was conducted by asking participants in both the intervention and control groups to wear sealed pedometers for 7 days at baseline, 6 months, and 12 months (between waking in the morning and going to bed at night, except during water-based activities).

#### Mediator Outcomes

Mediator outcomes were collected at baseline and 6 months via a self-reported questionnaire and included planning [38], self-determined motivation (ie, identified regulation, integrated regulation, and intrinsic motivation) [39,40], habit [41], recovery and maintenance self-efficacy [42], outcome satisfaction [43,44], social norms [45], and workplace norms [45]. These constructs were measured as they are central to the behavior change theories upon which the intervention was designed and represented the assumed pathways through which the intervention was hypothesized to lead to a change in physical activity behavior [17]. Exploring whether engagement with the various components of the intervention was related to changes in these constructs is useful to identify the *active ingredients* of the intervention, determine the degree to which the intervention worked as intended, and improve our understanding of how the intervention led to a change in behavior. Self-reported questionnaire data were collected via the Web-based platform Qualtrics (Qualtrics, Provo, Utah, USA).

#### Predictors of Nonusage Attrition

Predictors of nonusage attrition were sociodemographic, mediator, and environmental variables (assessed by questionnaire) and physical activity measures (pedometer steps per day) collected at baseline. Sociodemographic variables included age, gender, highest educational level, income, marital status, and self-reported height and weight (used to compute body mass index). Measures of health included Short Form-8 physical and mental health component scores [46], the Quality of Life health state utility measure and weighted health index [47], and the Warwick-Edinburgh Mental Wellbeing Scale [48,49]. Mediator variables included outcome expectations [43], physical activity self-efficacy [50], intention [51], planning [38], financial motivation [52,53], self-determined motivation (ie, identified regulation, integrated regulation, and intrinsic motivation) [39,40], habit [41], recovery and maintenance self-efficacy [42], outcome satisfaction [43,44], and social norms and workplace norms [45]. Perceptions of workplace environment (attractiveness, safety, accessibility, and availability) were also collected at baseline [54]. Descriptions of assessed variables are provided in Multimedia Appendix 2.

### Statistical Analysis

These analyses are exploratory and should be interpreted with caution because of multiple testing. The level of significance was *P*<.05 for all analyses. Analyses were carried out using Stata 13 (StataCorp) [55]. All questionnaire items were coded so that higher numerical variables equaled higher values of the construct.

#### Objective 1: To Determine Whether Levels of Engagement in Different Components of the Intervention Predicted Physical Activity Measured 6 Months Post Baseline for Participants Assigned to the Intervention Group

Random-effects generalized least-squares regressions were run with 6-month physical activity (ie, pedometer steps per day) as the dependent variable and engagement variables (ie, percentage of intervention days in which participants undertook at least 10 min of physical activity captured using the PAL scheme physical activity monitoring system, percentage of intervention weeks participants logged onto the PAL website, percentage of earned points redeemed, frequency of hits on each of the 6 website intervention components for every 10 days the participant accessed the website, and total number of website sections accessed at least once) as the independent variables. The model was adjusted for randomization stratum (large>50, medium=20-50, small<20 or schools or colleges), season (6-month follow-up occurred between December 2015 and April 2016 versus 6-month follow-up occurred between July 2016 and August 2016), and baseline pedometer steps per day with SEs and *P* values adjusted for clustering (3 clusters based on size and 1 cluster for educational establishments). Random-effects models explicitly modeled the dependence between observations within the same cluster by including the random effect. This represented the amount by which the intercept for a given cluster differed from the overall mean intercept value [56]. These analyses were conducted using Stata’s *xtreg* command with the *vce (cluster)* option specified. Engagement variables showing a significant relationship with 6-month physical activity in univariable analyses (*P*<.05) were included in a multivariable model with backward elimination of the predictor with the highest *P* value until all included predictors had *P*<.05. This determined the combined effects of all relevant predictors on 6-month physical activity. The distributions of residuals for each regression were plotted to check for normality. Partial regression plots were used to identify influential points, and homogeneity of variances was checked by graphing residual versus fitted values.

This paper focused on pedometer steps per day collected at 6 months only as the primary study outcomes were collected at 6 months, the intervention period was 6 months, and the significant negative intervention effect observed for pedometer steps per day had dissipated at 12 months [4].

#### Objective 2: To Determine Whether Levels of Engagement in Different Components of the Intervention Predicted Psychosocial Variables (ie, Mediators) Targeted by the Intervention at 6 Months Post Baseline

Random-effects generalized least-squares regressions were run with 6-month mediators as the dependent variable and engagement variables (ie, percentage of intervention days in which participants undertook at least 10 min of physical activity captured using the PAL scheme physical activity monitoring system, percentage of intervention weeks participants logged onto the PAL website, percentage of earned points redeemed, frequency of hits on each of the 6 website intervention components for every 10 days the participant accessed the website, and total number of website sections accessed at least once) as the independent variables. These analyses used the same procedures outlined under Objective 1 and additionally included baseline values of the relevant mediator as a covariate.

#### Objective 3: To Investigate Rates of Nonusage Attrition for Participants Recording Daily Activity via the Physical Activity Loyalty Scheme Physical Activity Monitoring System and Logging onto the Physical Activity Loyalty Scheme Website, and Baseline Predictors (ie, Sociodemographic, Mediator, Environmental, and Physical Activity Variables) of Nonusage Attrition for Participants in the Intervention Group

Survival curves for time to nonusage attrition were plotted separately for participants’ use of the physical activity monitoring system to record daily activity and website use. The median usage (ie, the time by which 50.0% of participants’ usage had lapsed; 211/422 for use of the physical activity monitoring system to record daily activity and 209/418 for logging onto the website) was then calculated. Baseline measures of sociodemographic variables, mediator variables, environmental variables, and physical activity were investigated as predictors of nonusage attrition of the physical activity monitoring system to record daily activity and nonusage attrition for use of the website using Cox proportional hazards regression analyses. In the first analysis, the time variable was the number of days until the first 2-week lapse from using the physical activity monitoring system to record daily activity. In the second analysis, the time variable was the number of days until the first 2-week lapse from logging onto the website. For each model, the event variable was coded 1 if nonusage attrition occurred or 0 if nonusage attrition did not occur. Univariable analyses were conducted on all predictor variables and those with *P*<.05 were included in a multivariable model with backward elimination of the predictor with the highest *P* value until all included predictors had *P*<.05. All analyses included SEs and *P* values corrected for clustering. The Efron procedure was used for handling ties as it is advocated over the Breslow method [57] and can be implemented with models adjusting SEs and *P* values for clustering. The proportional hazards assumption was tested for each model formally using the Schoenfeld residuals (*P*<.05 provided evidence to reject the proportional hazards assumption), and by visual inspection of scaled Schoenfeld residual plots [58]. Plots of −log(−log[survival]) versus log(time) were created for categorical predictors with nonparallelism indicating violation of the proportional hazards assumption.

As a sensitivity analysis for our definition of nonusage attrition, we repeated these analyses defining nonusage attrition as occurring if a participant had a 1-month (ie, 30 days) lapse from use.

## Results

### Baseline Characteristics

A total of 457 participants from 19 clusters were randomized to the intervention group. Baseline characteristics are reported in Multimedia Appendix 2.

### Engagement, Physical Activity, and Mediator Outcomes at 6 Months

Table 1 shows the 6-month engagement and nonusage attrition measures. The mean percentage of intervention days during which participants were recorded being active via the physical activity monitoring system was 24.7% (SD 21.8%; approximately 44/180 days), and the mean number of intervention weeks that participants logged onto the study website was 37.8% (SD 32.5%; approximately 9/24 weeks). Participants redeemed 39.3% (SD 42.5%; approximately 39 points for every 100 points earned) of their earned points on average. Participants clicked on 4 of the 6 website components at least once on average, and the component accessed with the highest frequency was monitoring and feedback. The 6-month physical activity and mediator outcomes are reported in Table 2.

**Table 1:** Descriptive statistics for 6-month engagement and nonusage attrition.

Variables		Statistics (6 months)	
		n	Mean (SD)
**Engagement**		
	Percentage of intervention days participants walked for at least 10 min captured via the physical activity monitoring system^a^	422	24.7 (21.8)
	Percentage of intervention weeks participants logged onto the website^b^	418	37.8 (32.5)
	Percentage of earned points redeemed^c^	422	39.3 (42.5)
	Frequency: Monitoring and feedback^d^	418	13.7 (3.5)
	Frequency: Rewards^d^	418	5.7 (4.5)
	Frequency: Maps^d^	418	3.4 (4.0)
	Frequency: Health information (physical activity)^d^	418	0.5 (1.7)
	Frequency: Health information (other)^d^	418	1.2 (3.2)
	Frequency: Discussion forums^d^	418	1.9 (4.2)
	Total number of sections (website)^e^	418	3.9 (1.5)
	Total minutes (recording daily activity via physical activity monitoring system)	422	1000 (987)
	Total minutes (PAL^f^ website)	418	1171 (2048)
**Nonusage attrition**		
	Days to nonusage attrition (recording daily activity via physical activity monitoring system)^g^	422	53.7 (61.2)
	Days to nonusage attrition (PAL website)^h^	418	31.7 (43.4)
	Number of participants with nonusage attrition for recording daily activity via physical activity monitoring system, n (%)	—^i^	375 (88.9)
	Number of participants with PAL website nonusage attrition, n (%)	—	403 (96.4)

^a^Percentage of days participants were recorded walking for at least 10 mins captured via the physical activity monitoring system.

^b^Percentage of weeks participants logged onto the website at least once.

^c^Percentage of total accumulated points which the participant had redeemed by 6 months.

^d^Frequency of hits (ie, total number of hits for every 10 days the participant accessed the website).

^e^Number of sections accessed on website at least once (0-6).

^f^PAL: Physical Activity Loyalty.

^g^Number of days until first 2-week lapse from recording daily activity via physical activity monitoring system.

^h^Number of days until first 2-week lapse from logging onto the website.

^i^Not applicable.

**Table 2:** Baseline and 6-month physical activity outcomes and scores on mediator variables.

Variables (scale range)	n	Baseline, mean (SD)	n	6 Months, mean (SD)
Physical activity self-efficacy (1-5)	439	2.91 (0.97)	—^a^	—
Intentions (1-7)	435	5.38 (1.68)	—	—
Outcome expectations (1-5)	418	3.37 (0.62)	—	—
Financial motivation (1-7)	439	1.71 (1.16)	—	—
Planning (1-4)	414	2.37 (0.69)	255	2.35 (0.74)
Social norms (1-7)	414	3.87 (1.20)	253	3.90 (1.13)
Identified regulation (1-5)	438	3.81 (0.87)	262	3.93 (0.82)
Integrated regulation (1-5)	439	3.12 (1.13)	258	3.41 (1.10)
Intrinsic motivation (1-5)	438	3.52 (0.99)	259	3.70 (0.91)
Habit (1-5)	437	2.89 (1.32)	256	3.18 (1.40)
Workplace norms (1-5)	439	3.20 (0.82)	260	3.19 (0.76)
Recovery self-efficacy (1-4)	438	2.36 (0.82)	261	2.41 (0.73)
Maintenance self-efficacy (1-4)	438	2.79 (0.86)	262	2.69 (0.83)
Outcome satisfaction (1-5)	404	3.85 (0.68)	257	3.87 (0.62)
Pedometer steps per day^b^	414	7977 (3602)	249	6990 (3078)

^a^Variable not measured at 6 months.

^b^12-month pedometer steps per day (mean 7790, SD 3462; n=210).

### Objective 1: To Determine Whether Levels of Engagement in Different Components of the Intervention Predicted Physical Activity Measured 6 Months Post Baseline for Participants Assigned to the Intervention Group

Table 3 shows the results of random-effects regressions with pedometer steps per day at 6 months as the dependent variable and use of specific intervention components as the independent variable, controlling for baseline pedometer steps per day, stratum, and season, with cluster-adjusted SEs and *P* values. Engagement variables that were significant predictors of 6-month pedometer steps per day in univariable analyses were included in a multivariable model that showed that the frequency of hits on the monitoring and feedback component of the website across the 6-month intervention period (*b*=50.2; SE=24.5; *P*=.04) and percentage of earned points redeemed across the 6-month intervention period (*b*=9.1; SE=3.3; *P*=.005) were positively related to 6-month pedometer steps per day, whereas the frequency of hits on the discussion forum component of the website across the 6-month intervention period (*b*=−69.3; SE=26.6; *P*=.009) was negatively related to 6-month pedometer steps per day. None of the other variables were significant predictors of 6-month pedometer steps per day in univariable analyses.

**Table 3:** Results of random-effects regressions with 6-month pedometer steps per day as the dependent variable and engagement indicators as independent variables among intervention group participants providing 6-month pedometer readings. Results are adjusted for stratum, season, and baseline pedometer steps per day with cluster-adjusted standard errors and *P* values

Engagement variables		Univariable models			Multivariable model^a^	
		n	*b* (SE)	*P* value^b^	n	*b* (SE)	*P* value^b^
**Engagement indicators**					
	Percentage of intervention days participants walked for at least 10 min captured via the physical activity monitoring system^c^	231	4.2 (8.5)	.62	—^d^	—	—
	Percentage of intervention weeks participants logged onto the website^e^	234	4.4 (6.0)	.47	—	—	—
	Percentage of earned points redeemed^f^	231	8.3 (4.1)	*.04*	230	9.1 (3.3)	*.005*
**Website sections**					
	Monitoring and feedback^g^	234	66.3 (18.5)	*<.001*	230	50.2 (24.5)	*.04*
	Rewards^g^	234	13.9 (36.0)	.70	—	—	—
	Maps^g^	234	−46.9 (43.7)	.28	—	—	—
	Health information: Physical activity^g^	234	34.9 (160.0)	.83	—	—	—
	Health information: Other^g^	234	25.2 (65.9)	.70	—	—	—
	Discussion forums^g^	234	−77.4 (27.1)	*.004*	230	−69.3 (26.6)	*.009*
	Number of sections^h^	234	−32.4 (117.4)	.78	—	—	—

^a^*R*-squared=0.54 for multivariable model. *R*-squared=0.51 for model including covariates only (ie, stratum, season, and baseline pedometer steps per day). Empty cells in this column show variables which were not included in the multivariable model.

^b^*P* values reported in italics show statistically significant results (*P*<.05).

^c^Percentage of days participants were recorded walking for at least 10 min captured via the physical activity monitoring system.

^d^Not applicable.

^e^Percentage of weeks participants logged onto the website at least once.

^f^Percentage of total accumulated points that the participant had redeemed by 6 months.

^g^Frequency of hits (ie, total number of hits for every 10 days the participant accessed the website).

^h^Number of sections accessed on website at least once (0-6).

### Objective 2: To Determine Whether Levels of Engagement in Different Components of the Intervention Predicted Psychosocial Variables (ie, Mediators) Targeted by the Intervention at 6 Months Post Baseline

The only mediator variable for which more than 1 independent variable was retained in the multivariable analysis was integrated regulation (Table 4). Engagement variables that were significant predictors of 6-month integrated regulation were included in a multivariable model that showed that the percentage of intervention days during which participants walked for at least 10 min captured via the PAL scheme physical activity monitoring system over the 6-month intervention period (*b*=0.008; SE=0.002; *P*<.001) and the frequency of hits on the monitoring and feedback component of the website across the 6-month intervention period (*b*=0.03; SE=0.01; *P*=.02) were positively related to 6-month pedometer steps per day, whereas the frequency of hits on the discussion forum component of the website across the 6-month intervention period (*b*=−0.02; SE=0.01; *P*=.02) was negatively related to 6-month pedometer steps per day. The results of all univariable analyses are presented in Multimedia Appendix 3.

**Table 4:** Results of multivariable random-effects regressions with 6-month integrated regulation as the dependent variable and engagement indicators as independent variables among intervention group participants providing 6-month data. Results are adjusted for stratum, season, baseline pedometer steps per day, and integrated regulation with cluster-adjusted standard errors and *P* values

Engagement variables		Univariable models			Multivariable model^a^	
		n	*b* (SE)	*P* value^b^	n	*b* (SE)	*P* value^b^
**Engagement indicators**					
	Percentage of intervention days participants walked for at least 10 mins captured via the physical activity monitoring system^c^	238	0.007 (0.002)	*.004*	236	0.008 (0.002)	*<.001*
	Percentage of intervention weeks participants logged onto the website^d^	240	0.004 (0.002)	*.02*	—^e^	—	—
	Percentage of earned points redeemed^f^	—	0.000 (0.001)	.82	—	—	—
**Website sections**					
	Monitoring and feedback^g^	240	0.03 (0.01)	*.02*	236	0.03 (0.01)	*.02*
	Rewards^g^	—	0.00 (0.01)	.97	—	—	—
	Maps^g^	—	−0.02 (0.01)	.10	—	—	—
	Health information: Physical activity^g^	240	0.11 (0.05)	*.03*	—	—	—
	Health information: Other^g^	—	0.06 (0.06)	.27	—	—	—
	Discussion forums^g^	240	−0.02 (0.01)	*.03*	236	−0.02 (0.01)	*.02*
	Number of sections^h^	240	0.09 (0.03)	*.005*	—	—	—

^a^*R*-squared=0.59 for multivariable model. *R*-squared=0.50 for model including covariates only (ie, stratum, season, baseline pedometer steps per day, and baseline integrated regulation). Empty cells in this column show variables which were not included in the multivariable model.

^b^*P* values reported in italics show statistically significant results (*P*<.05).

^c^Percentage of days participants were recorded walking for at least 10 mins captured via the physical activity monitoring system.

^d^Percentage of weeks participants logged onto the website at least once.

^e^Not applicable.

^f^Percentage of total accumulated points that the participant had redeemed by 6 months.

^g^Frequency of hits (ie, total number of hits for every 10 days the participant accessed the website).

^h^Number of sections accessed on website at least once (0-6).

### Objective 3: To Investigate rates of Nonusage Attrition for Participants Recording Daily Activity Via the Physical Activity Loyalty Scheme Physical Activity Monitoring System and Logging onto the Physical Activity Loyalty Scheme Website, and Baseline Predictors (ie, Sociodemographic, Mediator, Environmental, and Physical Activity Variables) of Nonusage Attrition for Participants in the Intervention Group

**Figure 1:**
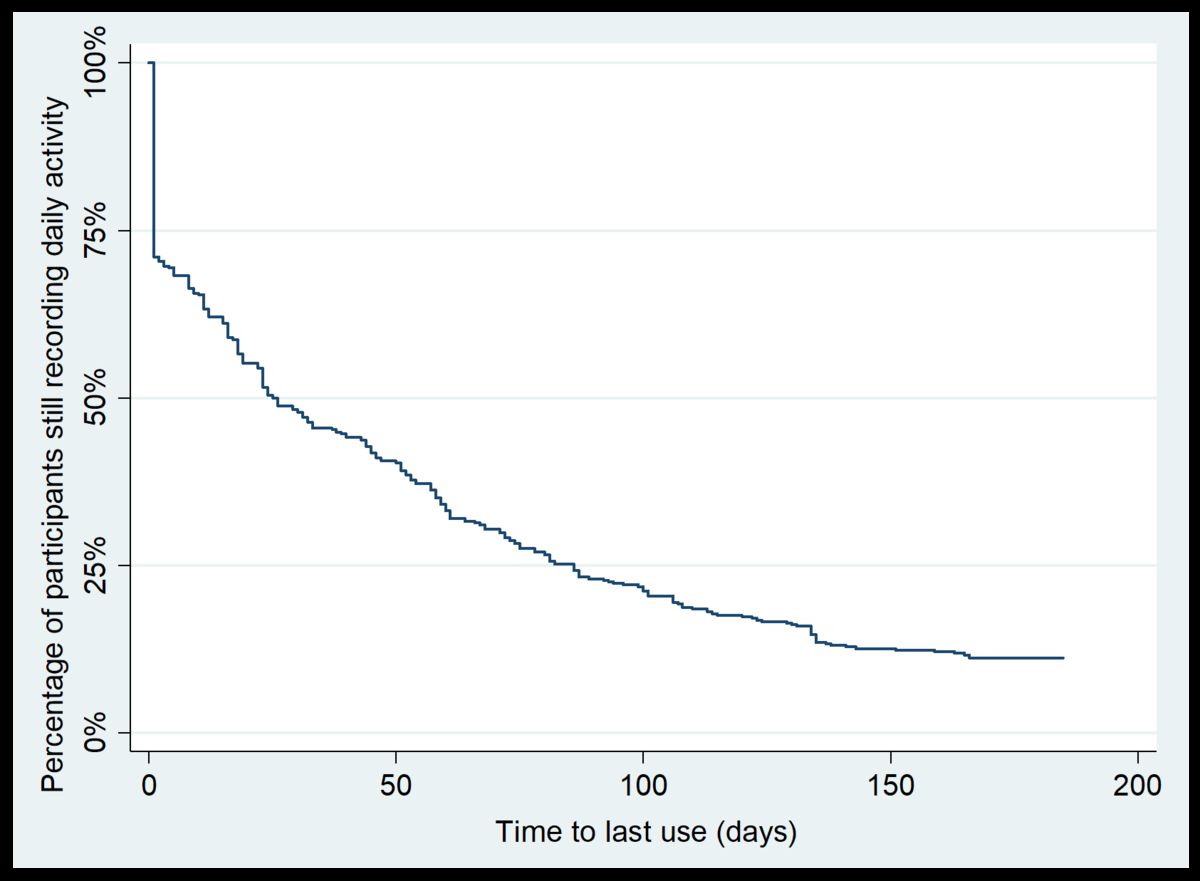
Survival curve for time to nonusage attrition for recording daily activity via the physical activity monitoring system (n=422).

**Figure 2:**
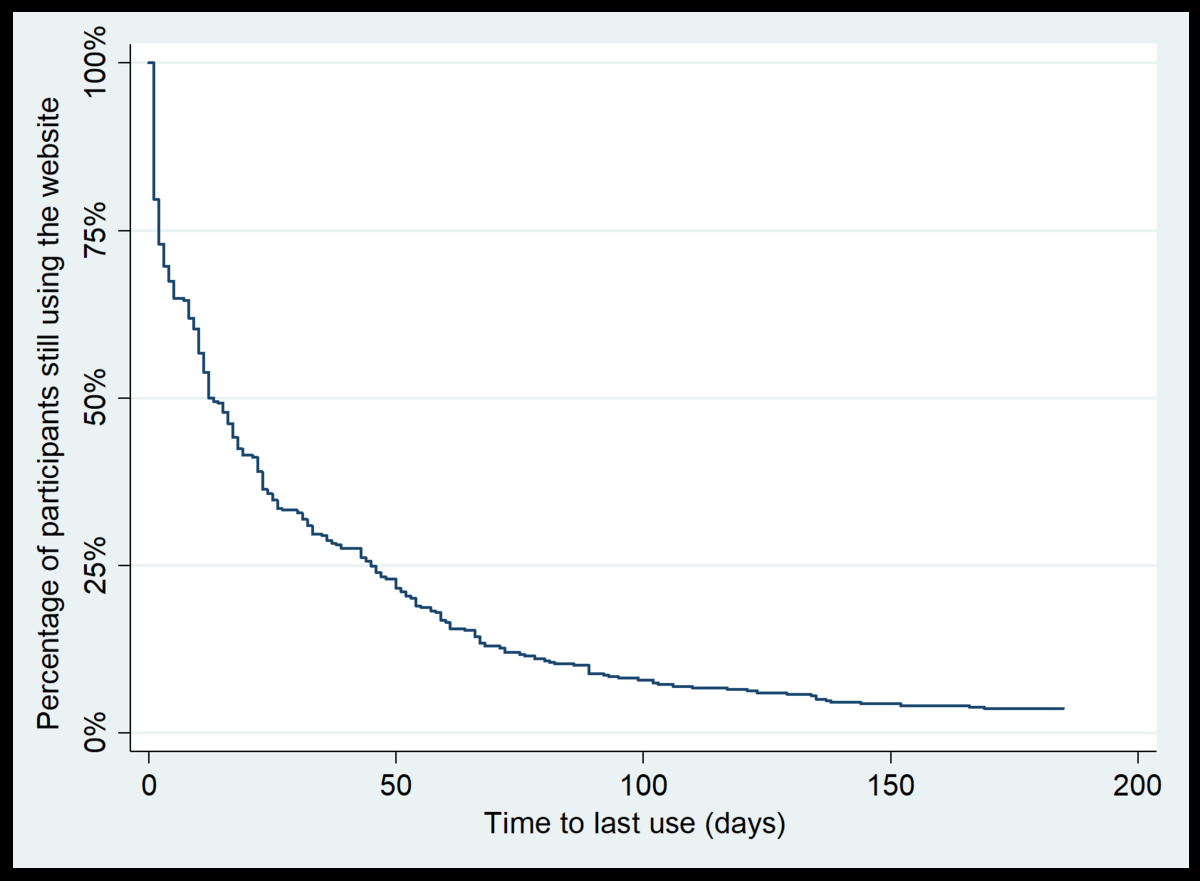
Survival curve for time to nonusage attrition for use of the website (n=418).

The median usage (ie, the time by which 50.0% of participants’ usage had lapsed) was 26 days for use of the physical activity monitoring system to record daily activity (nonusage attrition occurred for 211/422 participants; Figure 1) and 13 days for use of the website (nonusage attrition occurred for 209/418 participants; Figure 2). Nonusage attrition of the physical activity monitoring system to record daily activity occurred for 88.9% of participants (375/422), and website nonusage attrition occurred for 96.4% of participants (403/418). In both figures, the vertical section of the curve indicates that there was a proportion of participants who did not use the intervention component within the first 2 weeks of the intervention period (approximately equal to 25.0%, or 106/422, of the intervention group for use of the physical activity monitoring system and approximately equal to 20.0%, or 84/418, of the intervention group for use of the website).

Univariable and multivariable Cox regression analyses are presented in Multimedia Appendix 4. The multivariable analysis for use of the physical activity monitoring system to record daily activity showed that having higher levels of identified regulation at baseline (hazard ratio [HR] 0.88, 95% CI 0.81-0.97) and having higher levels of recovery self-efficacy at baseline (HR 0.88, 95% CI 0.80-0.98) reduced the risk of attrition. In contrast, having a higher perception of the safety of the workplace environment for physical activity at baseline (HR 1.07, 95% CI 1.02-1.11) was associated with a higher risk of attrition. The multivariable analysis for website use showed that having higher values on the EuroQoL weighted health index (HR 0.33, 95% CI 0.12-0.91), having higher levels of financial motivation at baseline (HR 0.93, 95% CI 0.87-0.99), or having a higher perception of the availability of physical activity opportunities in the workplace environment at baseline (HR 0.96, 95% CI 0.93-0.99) reduced the risk of attrition. Formal tests and visual inspection of plots showed no evidence for violation of the proportional-hazards assumption for the multivariable models. The results of the sensitivity analysis for our definition of nonusage attrition repeating these analyses with nonusage attrition defined as occurring if a participant had a 1-month (ie, 30 days) lapse from use have been reported in Multimedia Appendix 5.

## Discussion

### Principal Findings

This study examined whether overall engagement (ie, using the physical activity monitoring system to record physical activity, accessing the study website, and redeeming earned points for financial rewards) in a 6-month workplace physical activity intervention (ie, the PAL scheme) and engagement with specific intervention components (ie, specific sections of the website) were associated with physical activity and mediator outcomes at 6 months. Time to nonusage attrition for different intervention components and predictors of nonusage attrition (ie, sociodemographic, mediator, environmental, and physical activity variables) were also investigated. Due to the nature of the wait-list control, there are no available data on intervention engagement and nonusage attrition for the control group. Therefore, we are cautious not to overinterpret the results and draw causal conclusions from these analyses. Multivariable generalized least-squares regression analyses revealed that higher levels of engagement with some intervention components were significantly related to 6-month pedometer steps per day. Several baseline predictors of nonusage attrition were also identified using the Cox proportional hazards regression analyses.

#### Intervention Engagement and Physical Activity

This study found that overall levels of engagement with the intervention (ie, using the physical activity monitoring system to record physical activity and accessing the study website) were not related to physical activity at 6 months in contrast to the findings of several previous studies [8,59]. A plausible explanation is that our indicators may not have sufficiently captured participants’ true levels of engagement. Time spent on the website was not included as a measure of engagement because of the unstructured nature of website access, which meant that participants may have been engaging in other activities when logged on. Previous Web-based intervention studies investigating engagement include the website as the main intervention component and require participants to spend a significant amount of time on the website [14,15,29,32,33]. In contrast, the website was a mode of intervention delivery for the PAL study (the main intervention components were the financial incentive, placing sensors in an outdoor environment, and self-monitoring). Therefore, the time spent on the website is less relevant as an indicator of intervention engagement in this study than for the previous studies. Instead, we assumed that a higher frequency of hits on a particular section of the website for every 10 days of website use indicated higher levels of interest (and willingness to engage) in that aspect of the intervention. However, Baltierra et al noted that even this may be problematic as it gives no indication of whether participants are reading and comprehending the information or merely clicking on the various sections [29]. This illustrates the complexity inherent in measuring engagement in behavior change interventions and the need for a standardized approach [60,61].

Examining intervention engagement as a whole may not be sufficient to explain physical activity behavior change for this intervention, given the observed decline in physical activity for intervention participants. In particular, the use of multiple or multicomponent engagement measurements is recommended to track participant engagement in all the components of complex interventions [8]. Therefore, we also examined whether engagement with different intervention components was related to physical activity. The self-monitoring and feedback component was the most frequently accessed aspect of the website, and a higher frequency of accessing it was associated with a significant increase in physical activity at 6 months. This finding may indicate that when participants focused more on the scheme’s self-monitoring and feedback aspects, this was associated with less of a decline in physical activity and is in line with the results of the study’s mediation analyses, which found that planning and habit formation are important mediators [5]. Previous research also shows that self-regulation techniques and self-monitoring are useful strategies for physical activity behavior change or weight loss [36,62-67], and 1 previous systematic review of Web-based interventions also highlights that Web-based self-monitoring is a potentially effective technique [68]. Redeeming a higher proportion of earned points for rewards was associated with slightly higher physical activity levels at 6 months. This finding expands upon the study’s mediation analyses, which found that financial motivation was not related to physical activity behavior at 6 months and proposed that it is possible that the participants did not find the financial incentives attractive enough to trigger behavior change in the first place [5]. It appears that when participants found the financial incentives desirable and redeemed their accumulated points, this was associated with less of a decline in physical activity. Previous studies have also shown that for financial incentives to be successful in inducing behavior change, the reward on offer must be deemed worthwhile to the individual participant. For example, monetary value [69] or type (eg, individual versus group based) [70] of reward can impact its effectiveness for behavior change.

A higher frequency of accessing the discussion forum component of the website was associated with a significant decline in physical activity at 6 months. Discussion forums were included on the PAL website as a means of providing social support for behavior change. For example, it was expected that participants would use these forums to contact researchers and interact with other participants to support behavior change. However, participants mainly used this component to make queries and report technological issues. This may indicate that participant frustration with some perceived limitations of the intervention, which were highlighted in a separate qualitative process evaluation [71], contributed to the overall negative impact on physical activity. A previous study finding negative intervention effects on physical activity behavior concluded that reduced support for the intervention over time was a contributing factor and cited similar reasons (eg, lack of variety in activities resulting in participant boredom and restrictions on the availability of time or space) [72].

#### Intervention Engagement and Mediator Outcomes

A higher frequency of accessing the website’s feedback and monitoring component was also associated with increases in integrated regulation (in addition to physical activity behavior), further highlighting the importance of this particular intervention component. For example, there is evidence from the self-determination theory that more intrinsically motivated behavior is more likely to be maintained as it fulfills the basic psychological needs for competence, autonomy, and relatedness to others [10]. In comparison, engagement with the financial incentive component of the intervention (ie, redeeming a higher proportion of earned incentives for rewards or a higher frequency of accessing the website’s rewards component) was not found to be related to the levels of identified regulation, integrated regulation, and intrinsic motivation. Thus, this study contributes unique evidence to contravene one of the main criticisms of financial incentives that is highlighted in the self-determination theory literature (ie, that the use of financial incentives should have a *crowding* *out* effect on intrinsic motivation for behaviors that are already internalized [73]). This paper provides further and supporting evidence for the findings of the study’s mediation analysis to suggest financial incentives do not necessarily diminish more internal forms of motivation when delivered as part of a complex multicomponent behavior change intervention [5].

#### Nonusage Attrition

Nonusage attrition for use of the physical activity monitoring system and use of the website was high as most participants lapsed from using one or both features. High levels of attrition are commonly observed for use of Web-based interventions [27,74,75]; however, program usage is generally expected to be higher for controlled trials compared with freely accessible programs as participants are more likely to be motivated and committed to taking part in the study [13]. A 2012 systematic review of Web-based interventions [27] found that approximately half of the participants adhere to interventions. The definition of nonusage attrition (ie, occurring at the time of the first 2-week lapse from intervention use) adopted in this study may have contributed to the high levels of attrition observed. Although other studies of Web-based physical activity interventions have adopted this definition, it may be less applicable to the analysis of nonusage attrition in workplace interventions for which a 2-week lapse from intervention use may occur if a participant is on annual leave or is absent from work for 2 weeks or more. Therefore, every 2-week lapse from intervention use may not indicate that the participant had intentionally ceased intervention use.

The results of this analysis are consistent with findings in previous intervention studies, showing that participants with higher reported health status at baseline (versus lower health status) [76] have decreased risk for nonusage. It has previously been observed that Web-based interventions are frequently not successful in reaching individuals for whom health behavior change is needed most (eg, those with lower health status) [77]. Participants who were more financially motivated, who had higher levels of identified regulation, or who had higher levels of recovery self-efficacy at baseline were at lower risk for nonusage in this study. Individuals with higher levels of financial motivation may have been encouraged to continue participation in the scheme over time to continue benefitting from financial rewards. There is evidence that identified regulation and recovery self-efficacy are constructs that are important for long-term behavioral maintenance [78-82]. Identified regulation refers to behavior that is freely enacted based on the perceived value of its outcomes to the individual [83]. Individuals with higher levels of identified regulation may have been encouraged to engage in more continuous use of the intervention (ie, recording daily activity via the physical activity monitoring system) to achieve these valued outcomes (eg, improved health). Recovery self-efficacy refers to the individual’s beliefs about their capability to return to physical activity following a lapse. Therefore, someone with higher levels of recovery self-efficacy has faith in their competence to regain control following a setback (ie, period of inactivity) [84]. It makes sense that individuals with higher recovery self-efficacy would experience a longer period of intervention usage before encountering their first 2-week lull because they are quicker to recover from a lapse.

Finally, perceptions of the workplace environment were shown to be related to nonusage risk in this study. As the intervention required participants to engage in physical activity in the outdoor environment of the workplace, it is plausible that their perceptions of the workplace environment with respect to physical activity may have influenced the use they made of the scheme. For example, an important component of the PAL scheme was the provision of information on opportunities for physical activity in the workplace environment, and intervention participants had access to maps on the study website marking out suggested walking routes. There is evidence that supportive social and physical environments facilitate behavior change maintenance by lowering the opportunity cost of behavior [85]. Taken together, these results indicate that it may be possible at baseline to identify those participants who are at the highest risk for nonusage attrition and to include strategies in the intervention design for retention.

### Strengths and Limitations

A strength of this study is the examination of engagement as separate individual indicators related to the different components (ie, behavior change techniques) of the intervention. Furthermore, previous studies have investigated whether engagement (or adherence) is predictive of behavioral outcomes [31,33,59,86] without consideration of how they may relate to psychosocial outcomes (ie, mediators) targeted by the intervention and thought to lead to behavior change. Nonusage attrition was assessed in relation to use of more than 1 intervention component (ie, use of the physical activity monitoring system and the website), and this improves upon previous studies that typically assessed nonusage attrition in relation to website use only [76,87-91].

Although previous research on engagement in intervention studies has compared engagement between an intervention arm and a comparison arm [15], we were unable to include comparable engagement data from our control group because of the nature of the waitlist control. Therefore, our analysis is limited to intervention group participants only, in line with the approach adopted in previous similar studies [92]. Our analysis is also limited to responders at 6 months (ie, those who provided pedometer steps per day and mediator measurements). Furthermore, although measures of the frequency of hits on different sections of the website may indicate the participant’s level of interest in a specific intervention component, they do not capture how well the participant processed the information. Another potential limitation is that only baseline variables were investigated as predictors of nonusage attrition. However, our goals were broadly similar to previous studies of predictors of nonusage attrition [90], the aim being to better describe the groups who will continue engaging in an intervention at enrollment. As previously discussed, our definition of nonusage attrition (ie, occurring at the time of the first 2-week lapse from intervention use) may have contributed to the high levels of nonusage attrition observed because of participants potentially taking a 2-week period of annual leave or other absence from work. Therefore, any period of nonuse may not have been indicative of an intentional lapse from using the intervention. However, provided such unintentional lapses did not occur differentially between groups, it can be reasonably assumed that the results of the survival analyses, indicating groups of participants who were at higher risk for nonuse, will not have been spuriously impacted. Future Web-based intervention studies implemented in workplace settings could improve the assessment of nonusage attrition by including a feature to capture whether the participant is present at the worksite.

### Implications for Future Research

Future intervention studies should measure levels of engagement and nonusage with a view to making recommendations for retaining groups of participants who are at the highest risk for nonusage and lack of intervention engagement. This is particularly important for studies of Web-based interventions that are known to be particularly susceptible to lack of participant engagement and high nonusage attrition [13]. Better guidelines on how to measure intervention engagement are needed. For example, although commonly used markers of engagement (eg, the number of hits on certain website pages and time spent on the website) may indicate greater interest in different intervention components, they do not capture how much information is absorbed and processed. Regular knowledge quizzes may be helpful in this regard, but even these measures are problematic (eg, it is unclear whether the results can be attributed to intervention delivery, the participant’s engagement, or another factor) [29]. Clearly, the idea of intervention engagement is complex and multifaceted [93,94]. Thus, its assessment should move beyond the utilization of simple metrics to incorporate user engagement patterns over time [93,95,96]. When attempting to define an intervention’s *intended* *use* and assessing adherence or engagement, researchers should refer extensively to the assumed working mechanisms of the intervention. This will aid the standardization of the concepts of intervention adherence and engagement, which are often underdeveloped and improperly used in the current literature [61,94]. The Medical Research Council guidance on developing and evaluating complex interventions currently makes no reference to intervention engagement and nonusage attrition [97], and their process evaluation guidelines refer to engagement in a general way [12]. How to measure and analyze engagement and nonusage in complex public health interventions is a key gap in the literature. Researchers should consider developing engagement and retention strategies tailored toward specific groups of participants identified as being at risk for low engagement and high levels of nonusage attrition. Such endeavors should make use of behavior change theory and behavior change techniques within intervention trials, using similar approaches to how interventions are currently designed for changing behavior.

Researchers could make use of baseline data to identify participants who are at risk of nonengagement and nonusage attrition and design specific strategies to combat this. The findings of this study suggest that researchers should explore ways to keep those participants who are in worse health engaged with interventions for their entire duration. Interventionists should consider how to retain participants who are initially less financially motivated in studies whose main component is the offer of financial rewards. For studies of physical activity interventions requiring behavioral practice in the outdoor environment (as was the case for this study), participants’ perceptions of the environment with respect to physical activity are important influencing factors that should be considered for engagement and nonusage.

### Conclusions

More frequent use of the self-monitoring and feedback components of the intervention website (ie, self-monitoring and feedback and goal setting) and the redemption of a higher proportion of earned points for financial rewards (ie, immediate reward contingent on behavior change) were associated with increases in physical activity at 6 months for intervention group participants in the PAL study. Conversely, more frequent use of website discussion forums (ie, social support) was associated with decreases in physical activity at 6 months. A possible explanation for the negative association of discussion forum use with 6-month physical activity was that rather than making use of these forums to build social support for physical activity, participants generally used them as platforms to make queries or raise concerns. Therefore, it appears that the decline in physical activity behavior at 6 months for intervention group participants was due, at least in part, to participant dissatisfaction with some perceived study limitations (eg, technical glitches and limited financial rewards and physical activity opportunities for which rewards could be earned), which emerged in a separate qualitative process evaluation [71]. Levels of intrinsic motivation were not associated with the percentage of rewards redeemed or with the frequency of accessing the reward component of the website. Therefore, in contrast to the hypothesis of self-determination theory that offering financial rewards may *crowd* *out* intrinsic motivation, our results support that intrinsic motivation is not necessarily diminished when rewards are offered as part of a complex multicomponent intervention (eg, a higher frequency of accessing some of the website’s other components was actually associated with higher intrinsic motivation). Rates of nonusage attrition were high, and survival analysis showed that participants who were in worse health at baseline were at higher risk for nonuse. Financial motivation, identified regulation, recovery self-efficacy, and perceptions of the environment were also risk factors for nonusage. Guidelines to measure engagement and improve nonusage attrition should be established and strategies incorporated into study design to ensure that participants adhere to interventions in their intended form.

## Appendix

Multimedia Appendix 1 Overview of trial procedures and the Physical Activity Loyalty intervention programme.

Multimedia Appendix 2 Description of assessed variables and baseline characteristics.

Multimedia Appendix 3 Results of univariable random-effects regressions with individual mediators as dependent variables and engagement indicators as independent variables.

Multimedia Appendix 4 Univariable and multivariable Cox regression analyses.

Multimedia Appendix 5 Univariable and multivariable Cox regression analyses defining nonusage attrition as occurring at the first lapse from use of 1 month or longer.

## Abbreviations

HR: hazard ratio
NI: Northern Ireland
NIHR: National Institute for Health Research
PAL: Physical Activity Loyalty

## References

[1] Andersen LB, Mota J, Di Pietro L (2016). Update on the global pandemic of physical inactivity. Lancet.

[2] (2010). Department of Health.

[3] Marcus B, Forsyth L (2009). Motivating people to be physically active.

[4] Hunter R, Gough A, Murray J, Tang J, Brennan S, Chrzanowski-Smith O, Carlin A, Patterson C, Longo A, Hutchinson W, Prior L, Tully M, French D, Adams J, McIntosh E, Xin Y, Kee F (2019). Do loyalty card schemes encourage physical activity? A cluster RCT. NIHR Journals Library.

[5] Hunter RF, Tully MA, Davis M, Stevenson M, Kee F (2013). Physical activity loyalty cards for behavior change: a quasi-experimental study. Am J Prev Med.

[6] Saul JE, Amato MS, Cha S, Graham AL (2016). Engagement and attrition in Internet smoking cessation interventions: insights from a cross-sectional survey of. Internet Interv.

[7] Saunders RP, Evans MH, Joshi P (2005). Developing a process-evaluation plan for assessing health promotion program implementation: a how-to guide. Health Promot Pract.

[8] Thomson JL, Tussing-Humphreys LM, Goodman MH, Zoellner JM (2015). Engagement indicators predict health changes in a lifestyle intervention. Am J Health Behav.

[9] Campbell M, Fitzpatrick R, Haines A, Kinmonth AL, Sandercock P, Spiegelhalter D, Tyrer P (2000). Framework for design and evaluation of complex interventions to improve health. Br Med J.

[10] Michie S, West R, Campbell R, Brown J, Gainforth H (2014). ABC of Behaviour Change Theories Book - An Essential Resource for Researchers, Policy Makers and Practitioners.

[11] Graham AL, Papandonatos GD, Cha S, Erar B, Amato MS, Cobb NK, Niaura RS, Abrams DB (2017). Improving adherence to smoking cessation treatment: intervention effects in a web-based randomized trial. Nicotine Tob Res.

[12] Moore G, Audrey S, Barker M, Bond L, Bonell C, Hardeman W, Moore L, O'Cathain A, Tinati T, Wight D, Baird J (2015). Process evaluation of complex interventions: Medical Research Council guidance. Br Med J.

[13] Eysenbach G (2005). The law of attrition. J Med Internet Res.

[14] Guertler D, Vandelanotte C, Kirwan M, Duncan MJ (2015). Engagement and nonusage attrition with a free physical activity promotion program: the case of 10,000 Steps Australia. J Med Internet Res.

[15] Kolt GS, Rosenkranz RR, Vandelanotte C, Caperchione CM, Maeder AJ, Tague R, Savage TN, Van Itallie A, Mummery WK, Oldmeadow C, Duncan MJ (2017). Using Web 2.0 applications to promote health-related physical activity: findings from the WALK 2.0 randomised controlled trial. Br J Sports Med.

[16] Blue CL, Black DR (2005). Synthesis of intervention research to modify physical activity and dietary behaviors. Res Theory Nurs Pract.

[17] Hunter RF, Brennan SF, Tang J, Smith OJ, Murray J, Tully MA, Patterson C, Longo A, Hutchinson G, Prior L, French DP, Adams J, McIntosh E, Kee F (2016). Effectiveness and cost-effectiveness of a physical activity loyalty scheme for behaviour change maintenance: a cluster randomised controlled trial. BMC Public Health.

[18] Miller N, Dollard J (1941). Social Learning and Imitation.

[19] Carver C, Scheier M (1981). Attention And Self-regulation: A Control-theory Approach To Human Behavior (springer Series In Social Psychology).

[20] Bandura A (1997). Self-Efficacy: The Exercise of Control.

[21] Deci E, Ryan R (1985). Intrinsic motivation and Self-determination in human behaviour.

[22] Haff N, Patel MS, Lim R, Zhu J, Troxel AB, Asch DA, Volpp KG (2015). The role of behavioral economic incentive design and demographic characteristics in financial incentive-based approaches to changing health behaviors: a meta-analysis. Am J Health Promot.

[23] Marteau TM, Ashcroft RE, Oliver A (2009). Using financial incentives to achieve healthy behaviour. Br Med J.

[24] Eysenbach G, CONSORT-EHEALTH Group (2011). CONSORT-EHEALTH: improving and standardizing evaluation reports of web-based and mobile health interventions. J Med Internet Res.

[25] World Health Organization (2010). Global recommendations on physical activity for health.

[26] Department Of Health.

[27] Kelders SM, Kok RN, Ossebaard HC, Van Gemert-Pijnen JE (2012). Persuasive system design does matter: a systematic review of adherence to web-based interventions. J Med Internet Res.

[28] van den Berg MH, Ronday HK, Peeters AJ, Voogt-van der Harst EM, Munneke M, Breedveld FC, Vliet Vlieland TP (2007). Engagement and satisfaction with an internet-based physical activity intervention in patients with rheumatoid arthritis. Rheumatology (Oxford).

[29] Baltierra NB, Muessig KE, Pike EC, LeGrand S, Bull SS, Hightow-Weidman LB (2016). More than just tracking time: complex measures of user engagement with an internet-based health promotion intervention. J Biomed Inform.

[30] Manwaring JL, Bryson SW, Goldschmidt AB, Winzelberg AJ, Luce KH, Cunning D, Wilfley DE, Taylor CB (2008). Do adherence variables predict outcome in an online program for the prevention of eating disorders?. J Consult Clin Psychol.

[31] Zbikowski SM, Hapgood J, Smucker Barnwell S, McAfee T (2008). Phone and web-based tobacco cessation treatment: real-world utilization patterns and outcomes for 11,000 tobacco users. J Med Internet Res.

[32] Couper MP, Alexander GL, Zhang N, Little RJ, Maddy N, Nowak MA, McClure JB, Calvi JJ, Rolnick SJ, Stopponi MA, Cole Johnson C (2010). Engagement and retention: measuring breadth and depth of participant use of an online intervention. J Med Internet Res.

[33] Glasgow RE, Christiansen SM, Kurz D, King DK, Woolley T, Faber AJ, Estabrooks PA, Strycker L, Toobert D, Dickman J (2011). Engagement in a diabetes self-management website: usage patterns and generalizability of program use. J Med Internet Res.

[34] Yu CH, Parsons JA, Mamdani M, Lebovic G, Hall S, Newton D, Shah BR, Bhattacharyya O, Laupacis A, Straus SE (2014). A web-based intervention to support self-management of patients with type 2 diabetes mellitus: effect on self-efficacy, self-care and diabetes distress. BMC Med Inform Decis Mak.

[35] Bassett DR, Ainsworth BE, Leggett SR, Mathien CA, Main JA, Hunter DC, Duncan GE (1996). Accuracy of five electronic pedometers for measuring distance walked. Med Sci Sports Exerc.

[36] Bravata DM, Smith-Spangler C, Sundaram V, Gienger AL, Lin N, Lewis R, Stave CD, Olkin I, Sirard JR (2007). Using pedometers to increase physical activity and improve health: a systematic review. J Am Med Assoc.

[37] Schneider PL, Crouter SE, Lukajic O, Bassett DR (2003). Accuracy and reliability of 10 pedometers for measuring steps over a 400-m walk. Med Sci Sports Exerc.

[38] Sniehotta Ff, Schwarzer R, Scholz U, Schüz B (2005). Action planning and coping planning for long-term lifestyle change: theory and assessment. Eur J Soc Psychol.

[39] Markland D, Tobin V (2004). A modification to the behavioural regulation in exercise questionnaire to include an assessment of amotivation. J Sport Exerc Psychol.

[40] Wilson P, Rodgers W, Loitz C, Scime G (2007). "It's who I am...really!" The importance of integrated regulation in exercise contexts. J Appl Biobehav Res.

[41] Verplanken B, Orbell S (2003). Reflections on past behavior: a self-report index of habit strength. J Appl Social Pyschol.

[42] Scholz U, Sniehotta F, Schwarzer R (2005). Predicting physical exercise in cardiac rehabilitation: the role of phase-specific self-efficacy beliefs. J Sport Exerc Psychol.

[43] Finch EA, Linde JA, Jeffery RW, Rothman AJ, King CM, Levy RL (2005). The effects of outcome expectations and satisfaction on weight loss and maintenance: correlational and experimental analyses--a randomized trial. Health Psychol.

[44] Rothman AJ, Sheeran P, Wood W (2009). Reflective and automatic processes in the initiation and maintenance of dietary change. Ann Behav Med.

[45] Ball K, Jeffery RW, Abbott G, McNaughton SA, Crawford D (2010). Is healthy behavior contagious: associations of social norms with physical activity and healthy eating. Int J Behav Nutr Phys Act.

[46] Ware J, Kosinski M, Dewey J, Gandek B (2001). How to score and interpret single-item health status measures: a manual for users of the of the SF-8 health survey.

[47] EuroQol Group (1990). EuroQol--a new facility for the measurement of health-related quality of life. Health Policy.

[48] Lloyd K, Devine P (2012). Psychometric properties of the Warwick-Edinburgh Mental Well-being Scale (WEMWBS) in Northern Ireland. J Ment Health.

[49] Tennant R, Hiller L, Fishwick R, Platt S, Joseph S, Weich S, Parkinson J, Secker J, Stewart-Brown S (2007). The Warwick-Edinburgh Mental Well-being Scale (WEMWBS): development and UK validation. Health Qual Life Outcomes.

[50] Marcus BH, Rossi JS, Selby VC, Niaura RS, Abrams DB (1992). The stages and processes of exercise adoption and maintenance in a worksite sample. Health Psychol.

[51] Fishbein M, Ajzen I (1975). Belief, Attitude, Intention, and Behavior: An Introduction to Theory and Research.

[52] Ryan R, Frederick C, Lepes D, Rubio N, Sheldon K (1997). Intrinsic motivation and exercise adherence. Int J Sport Psychol.

[53] Moller AC, McFadden HG, Hedeker D, Spring B (2012). Financial motivation undermines maintenance in an intensive diet and activity intervention. J Obes.

[54] Ogilvie D, Mitchell R, Mutrie N, Petticrew M, Platt S (2008). Perceived characteristics of the environment associated with active travel: development and testing of a new scale. Int J Behav Nutr Phys Act.

[55] StataCorp (2013). Stata.

[56] Stevens A (2001). The advanced handbook of methods in evidence based healthcare.

[57] Borucka J (2014). Methods for handling tied events in the Cox proportional hazard model. Studies of Oeconomica Posnaniensia.

[58] Schoenfeld D (1982). Partial residuals for the proportional hazards regression model. Biometrika.

[59] Heesch KC, Mâsse LC, Dunn AL, Frankowski RF, Mullen PD (2003). Does adherence to a lifestyle physical activity intervention predict changes in physical activity?. J Behav Med.

[60] Graham L, Wright J, Walwyn R, Russell AM, Bryant L, Farrin A, House A (2016). Measurement of adherence in a randomised controlled trial of a complex intervention: supported self-management for adults with learning disability and type 2 diabetes. BMC Med Res Methodol.

[61] Sieverink F, Kelders SM, van Gemert-Pijnen JE (2017). Clarifying the concept of adherence to eHealth technology: systematic review on when usage becomes adherence. J Med Internet Res.

[62] Hobbs N, Godfrey A, Lara J, Errington L, Meyer TD, Rochester L, White M, Mathers JC, Sniehotta FF (2013). Are behavioral interventions effective in increasing physical activity at 12 to 36 months in adults aged 55 to 70 years? A systematic review and meta-analysis. BMC Med.

[63] Abraham C, Graham-Rowe E (2009). Are worksite interventions effective in increasing physical activity? A systematic review and meta-analysis. Health Psychol Rev.

[64] Bird EL, Baker G, Mutrie N, Ogilvie D, Sahlqvist S, Powell J (2013). Behavior change techniques used to promote walking and cycling: a systematic review. Health Psychol.

[65] Michie S, Abraham C, Whittington C, McAteer J, Gupta S (2009). Effective techniques in healthy eating and physical activity interventions: a meta-regression. Health Psychol.

[66] Williams SL, French DP (2011). What are the most effective intervention techniques for changing physical activity self-efficacy and physical activity behaviour--and are they the same?. Health Educ Res.

[67] Bennett GG, Steinberg D, Askew S, Levine E, Foley P, Batch BC, Svetkey LP, Bosworth HB, Puleo EM, Brewer A, DeVries A, Miranda H (2018). Effectiveness of an app and provider counseling for obesity treatment in primary care. Am J Prev Med.

[68] Carolan S, Harris PR, Cavanagh K (2017). Improving employee well-being and effectiveness: systematic review and meta-analysis of web-based psychological interventions delivered in the workplace. J Med Internet Res.

[69] Finkelstein EA, Linnan LA, Tate DF, Birken BE (2007). A pilot study testing the effect of different levels of financial incentives on weight loss among overweight employees. J Occup Environ Med.

[70] Kullgren JT, Troxel AB, Loewenstein G, Asch DA, Norton LA, Wesby L, Tao Y, Zhu J, Volpp KG (2013). Individual- versus group-based financial incentives for weight loss: a randomized, controlled trial. Ann Intern Med.

[71] Gough A, Prior L, Kee F, Hunter RF (2018). Physical activity and behaviour change: the role of distributed motivation. Crit Public Health.

[72] Azevedo LB, Burges Watson D, Haighton C, Adams J (2014). The effect of dance mat exergaming systems on physical activity and health-related outcomes in secondary schools: results from a natural experiment. BMC Public Health.

[73] Promberger M, Marteau TM (2013). When do financial incentives reduce intrinsic motivation? Comparing behaviors studied in psychological and economic literatures. Health Psychol.

[74] Brindal E, Hendrie GA, Freyne J, Noakes M (2018). Incorporating a static versus supportive mobile phone app into a partial meal replacement program with face-to-face support: randomized controlled trial. JMIR Mhealth Uhealth.

[75] Van der Mispel C, Poppe L, Crombez G, Verloigne M, De Bourdeaudhuij I (2017). A self-regulation-based eHealth intervention to promote a healthy lifestyle: investigating user and website characteristics related to attrition. J Med Internet Res.

[76] Jahangiry L, Shojaeizadeh D, Montazeri A, Najafi M, Mohammad K, Yaseri M (2014). Adherence and attrition in a web-based lifestyle intervention for people with metabolic syndrome. Iran J Public Health.

[77] Verheijden MW, Jans MP, Hildebrandt VH, Hopman-Rock M (2007). Rates and determinants of repeated participation in a web-based behavior change program for healthy body weight and healthy lifestyle. J Med Internet Res.

[78] Deci E, Ryan R (1985). Intrinsic motivation and self-determination in human behavior.

[79] Deci E, Ryan R (2008). Facilitating optimal motivation and psychological well-being across life's domains. Can Psychol.

[80] Ryan R (2009). University of BATH.

[81] Schwarzer R (1992). Self-efficacy in the adoption and maintenance of health behaviors: theoretical approaches and a new model. Self-efficacy Thought Control action.

[82] Schwarzer R (2008). Modeling health behavior change: how to predict and modify the adoption and maintenance of health behaviors. Appl Psychol.

[83] Deci EL, Eghrari H, Patrick BC, Leone DR (1994). Facilitating internalization: the self-determination theory perspective. J Pers.

[84] Luszczynska A, Schwarzer R (2003). Planning and self-efficacy in the adoption and maintenance of breast self-examination: a longitudinal study on self-regulatory cognitions. Psychol Health.

[85] Kwasnicka D, Dombrowski SU, White M, Sniehotta F (2016). Theoretical explanations for maintenance of behaviour change: a systematic review of behaviour theories. Health Psychol Rev.

[86] Davies C, Corry K, Van Itallie A, Vandelanotte C, Caperchione C, Mummery WK (2012). Prospective associations between intervention components and website engagement in a publicly available physical activity website: the case of 10,000 Steps Australia. J Med Internet Res.

[87] Vandelanotte C, Kolt GS, Caperchione CM, Savage TN, Rosenkranz RR, Maeder AJ, Van Itallie A, Tague R, Oldmeadow C, Mummery WK, Duncan MJ (2017). Effectiveness of a Web 2.0 intervention to increase physical activity in real-world settings: randomized ecological trial. J Med Internet Res.

[88] Murray E, White IR, Varagunam M, Godfrey C, Khadjesari Z, McCambridge J (2013). Attrition revisited: adherence and retention in a web-based alcohol trial. J Med Internet Res.

[89] Wanner M, Martin-Diener E, Bauer G, Braun-Fahrländer C, Martin BW (2010). Comparison of trial participants and open access users of a web-based physical activity intervention regarding adherence, attrition, and repeated participation. J Med Internet Res.

[90] Neve MJ, Collins CE, Morgan PJ (2010). Dropout, nonusage attrition, and pretreatment predictors of nonusage attrition in a commercial Web-based weight loss program. J Med Internet Res.

[91] Lim S, Norman R, Clifton P, Noakes M (2014). Weight loss and attrition in overweight and obese young women during a 36-week internet-based lifestyle intervention. J Obes Weight Loss Ther.

[92] Mattila E, Lappalainen R, Välkkynen P, Sairanen E, Lappalainen P, Karhunen L, Peuhkuri K, Korpela R, Kolehmainen M, Ermes M (2016). Usage and dose response of a mobile acceptance and commitment therapy app: secondary analysis of the intervention arm of a randomized controlled trial. JMIR Mhealth Uhealth.

[93] Taki S, Lymer S, Russell CG, Campbell K, Laws R, Ong K, Elliott R, Denney-Wilson E (2017). Assessing user engagement of an mHealth intervention: development and implementation of the Growing Healthy App Engagement Index. JMIR Mhealth Uhealth.

[94] Perski O, Blandford A, West R, Michie S (2017). Conceptualising engagement with digital behaviour change interventions: a systematic review using principles from critical interpretive synthesis. Transl Behav Med.

[95] Paz Castro R, Haug S, Filler A, Kowatsch T, Schaub MP (2017). Engagement within a mobile phone-based smoking cessation intervention for adolescents and its association with participant characteristics and outcomes. J Med Internet Res.

[96] Kim JY, Wineinger NE, Taitel M, Radin JM, Akinbosoye O, Jiang J, Nikzad N, Orr G, Topol E, Steinhubl S (2016). Self-monitoring utilization patterns among individuals in an incentivized program for healthy behaviors. J Med Internet Res.

[97] Craig P, Dieppe P, Macintyre S, Michie S, Nazareth I, Petticrew M Medical Research Council.

