# Supplementary material for: Predicting Outcomes from Engagement With Specific Components of an Internet-Based Physical Activity Intervention With Financial Incentives: Process Analysis of a Cluster Randomized Controlled Trial
Source: J Med Internet Res. 2019 Apr 19;21(4):e11394. doi: 10.2196/11394 (PMC6498305; doi:10.2196/11394)
Supplement: Multimedia Appendix 1 [file jmir_v21i4e11394_app1.docx]

**Multimedia Appendix 1. Overview of trial procedures and the Physical Activity Loyalty intervention programme.**

#### Recruitment of Workplaces

The study targeted public sector office-based employees whose workplace was within Belfast or Lisburn city centres, Northern Ireland. Public sector organisations were purposively sampled from those within a 2 km radius of the city centre or which had opportunities for physical activity within a 2 km radius of their location, and had a minimum of 100 employees in predominantly office-based occupations. Meetings were held with senior management of these organisations to explain the purpose of the study and the practicalities. Workplaces were recruited between September 2014 and August 2015 and participant recruitment took place between January 2015 and October 2015.

#### Recruitment of Participants

Recruitment methods included email invitations to employees and posters placed around each workplace advertising the study. Emails and posters included the website address and a web-link was added to the organisations’ intranet sites (previously tested in the pilot study) [3]. Potential participants were able to access further information (including the Participant Information Sheet) and register their interest to participate on the study website. Interested individuals were asked to complete a screening questionnaire via the study website or by telephone, to confirm their eligibility, based on the following inclusion criteria: based at recruited worksite at least four hours/day (within core hours of 8 am-6 pm) on at least three days/week; current contract anticipated to last for the duration of the study (i.e. to exclude temporary workers); access to internet at work; able to give informed consent; able to communicate in English; no self-reported recent history of myocardial infarction or stroke or physical limitations that would limit ability to participate in physical activity (assessed using the Physical Activity Readiness Questionnaire). All individuals who met the eligibility criteria and consented to participate were contacted by a member of the research team by telephone or email to complete the baseline assessment. After the baseline assessment, clusters of participants were randomised to the intervention or control group using computer generated random numbers. Clusters were defined as the smallest organisational unit (e.g. a department or office/floor) within each participating workplace.

#### Sample

During recruitment, a revised power calculation was performed (with the approval of the funding body) assuming a less demanding effect size than in the study's original protocol. This was proposed in light of more recent literature published [4].

In the original protocol for the trial, the sample size calculation was determined using an anticipated effect size of d=0.21 which was based upon a previous meta-analysis of workplace based physical activity interventions. However, none of the studies which were included in this meta-analysis were incentive-based interventions for physical activity behaviour change. More recent literature has been published [4,5] including a meta-analysis showing a mean effect size of approximately 1600 steps (d=0.40). Additionally, the TRial of Economic Incentives to Promote Physical Activity (TRIPPA) study [4], which examined the influence of financial incentives on the effectiveness of a wireless-upload pedometer to encourage weekly physical activity goals, was powered to detect a difference of a minimum of 30 minutes of moderate-vigorous intensity physical activity (MVPA)/week between groups and reflective of a considerably higher effect size than assumed in the original calculation.

Therefore, the power calculation was updated as follows: For an effect size of 0.40, a study of 330 per group (or 660 in total) would have 90% power at the 5% significance level. Assuming a 15% drop-out, the study would need to randomise 776 participants.

### Randomisation, Concealment & Blinding

Clusters were the smallest work groups or units (e.g. a large open plan office) within each participating organisation. A random allocation sequence was drawn up by the trial statistician and group allocation was stratified to ensure a similar number of clusters in both Intervention and control groups. Research staff were blinded to group allocation until after data collection was completed. The outcome of the randomisation was communicated to participants by email after the baseline assessment.

#### Intervention Group

The PAL Scheme is a complex multi-component intervention based on concepts similar to those that underpin a high-street loyalty card aimed at encouraging repeated behaviour (i.e. loyalty) and includes a range of behaviour change techniques. Components include the provision of ‘points’ and rewards (financial incentives) contingent on meeting targeted physical activity behaviour goals (extrinsic motivation, goal-setting). Participants were encouraged to undertake 150 mins/week of physical activity which is in line with current guidelines. The PAL Scheme integrated a novel physical activity remote tracking system with web-based monitoring and evidence-based behaviour change tools (i.e. self-monitoring, goal-setting). The intervention was designed in line with the Medical Research Council (MRC) guidelines [6].

The six month intervention included placing of sensors (wifi beacons) in the vicinity of participating workplaces at specific locations to encourage physical activity within a 2 km radius of participants’ worksites (i.e. including the provision of prompts/cues to facilitate habit formation). The wifi beacons were placed at locations along footpaths, in local parks, leisure centres, shopping malls, bus stops and train stations. Maps of various walking routes and details about physical activity opportunities tailored to the workplace were provided on the study website (i.e. instruction on how to perform behaviour). Participant’s activity was logged when they passed within an approximate 25 m radius of the wifi sensors with their PAL keyfob when undertaking physical activity (e.g. walking). This logged the place, date and time of the bout of physical activity. Participants could log onto their account on the study website and receive real-time feedback on the number of minutes of physical activity logged by the tracking system. Minutes were converted to ‘points’ (ten ‘points’ for one minute of activity recorded), and collected ‘points’ were redeemable for rewards (downloadable retail vouchers) sponsored by, and redeemable at, local businesses. To reduce the risk of ‘gaming’, a daily ‘points’ cap was implemented and the transit times between sensors checked for anomalous values. Bonus rewards and ‘Double Points Days’ were offered when participants met specific weekly physical activity targets.

To determine incentive levels, stated preferences derived from Discrete Choice Experiments were used to assess mean Willingness to Accept, Willingness to Pay and the trade-offs participants would make for the attributes of the incentive programme. This information helped determine the level of the rewards available for earned ‘points’. In addition to the financial incentive element, the intervention had several other components designed to enhance the effectiveness of the incentives. These components were delivered via the study website and designed to have multiple effects: (a) to increase usage of the study website, (b) as effective behaviour change techniques in their own right, and (c) as techniques designed to aid the transition from more extrinsically motivated behaviour to more intrinsically motivated habitual behaviour. The techniques included the provision of regular tailored motivational emails, tailored feedback, information on walking routes in the vicinity of the participating workplaces and links to other resources such as physical activity advice and healthy eating guidelines. Self-regulation techniques such as goal setting, self-monitoring, and prompts to behaviour were also included.

*Underpinning theoretical framework:* The financial reward component of the intervention was based on principles of Learning Theory [7] by providing an immediate reward (extrinsic motivation) for behaviours that offer health gains in the future. It also contained elements based on other approaches, such as goal setting, prompts, self-monitoring, and habit formation which fit within a self-regulation control theory framework [8], motivational messages (persuasion), and social support (vicarious experience) which should increase self-efficacy according to Social Cognitive Theory [9]. Social Cognitive Theory also holds that satisfaction with the consequences of behaviour change can act as a reinforcing mechanism, in addition to the reinforcement of financial rewards. Thus, the financial incentive component was embedded in a complex intervention containing evidence-informed behaviour change techniques. Rewards were phased (i.e. offered less frequently in the last three months of the intervention) to reduce the emphasis on extrinsic motivation and increase the emphasis on intrinsic factors [10,11] according to Self-Determination Theory [12]. Figure 1.1 shown below presents the logic model underpinning the intervention development. Figures 1.2 and 1.3 show diagrams representing how the intervention worked.

#### Control Group

Participants assigned to the waiting-list control group were offered the opportunity to participate in the PAL scheme after the 12 month follow-up period. Participants in this group completed outcome measures at the same time points as the intervention group.

###

### Ethics and Consent

Ethical approval was sought and granted from the Office of Research Ethics Committees Northern Ireland (ORECNI) prior to the start of the study (Reference: 14/NI/0090). Fully informed consent was obtained from all participants prior to their inclusion in the study. Research Governance approval was granted from the South Eastern Health and Social Care Trust and Belfast Health and Social Care Trust.

**Deviations of the evaluation from the original protocol**

On 31/08/2015 the research team corresponded with the NIHR Public Health Research secretariat to indicate that the initial recruitment target was not met, explaining a variety of reasons why the original target proved problematic. Significant re-structuring of a number of the public sector organisations that were to host the intervention severely impacted on participant recruitment, and the study team undertook various actions to mitigate the shortfall (e.g. by extending the number of sites; and taking advice from Patient and Public Involvement (PPI) members on the Trial Steering Committee regarding alternative recruitment methods within the host organisations).

As recruitment numbers were lower than anticipated from the initial baseline data collection phase in Lisburn (from the re-structured local authority and healthcare Trust), the intervention phase started later than scheduled, in May 2014. It was decided that recruitment would continue across other agreed sites in Belfast {Queen's University Belfast (offering a pool of approx. 3000 staff) and the Stormont Civil Service Estate (offering a pool of approx. 3000 staff)} in order to attempt to meet the recruitment target and the intervention would be implemented on a rolling basis thereafter. Therefore, randomisation and the intervention phases were implemented in July and August 2015 in the Queen's University campus and the Stormont Estate, respectively.

Recruitment continued to be slower than anticipated, even with the addition of the new sites at Queen's University Belfast and the Stormont Estate. This was due to unforeseen circumstances (austerity related re-structuring of local authorities and within the civil service) and therefore approval was sought to recruit from two final sites within the Belfast Health and Social Care Trust (Royal Victoria Hospital and Belfast City Hospital). There followed an intense recruitment period in early September 2015. Participant recruitment was completed in January 2016 following the addition of a new worksite (total n=853).

A revised power calculation was undertaken, which took account of more recent literature on effect size estimates and the baseline data on cluster size, cluster variation and intra-class correlation co-efficient (ICC).

Further, in order to maximise trial retention, all participants received a £10 gift card at the six and 12 month stage for full completion of study outcomes (funded from internal sources). The trial was originally designed to include follow-up of participants to 18 months post-intervention. However, with unavoidable delays in initial recruitment attendant upon the re-structuring of the local authorities and in light of the findings at the six month follow up (i.e. significant decreases in the primary outcome for intervention group participants compared to controls), it was agreed with the study's funding body that follow-up should stop at 12 months rather than 18 months as originally planned.

**Recruitment**

A total of 1,209 employees expressed an interest in participating in the study and were assessed for eligibility. Reasons for exclusion included: not based at recruited worksite at least four hours/day on at least three days/week (n=93); current contract did not last the duration of the study (n=1); no longer wished to participate pre-randomisation (n=107); did not provide baseline data (n=150); unable to contact (n=5).

Eight hundred and fifty three individuals were randomized to either the Intervention (n=457) or the control group (n=396). The study flow diagram is presented in Figure 1.4 below.

**Withdrawals**

Participants noted as withdrawals were those who formally, via email, telephone, or in person, expressed their wish to no longer take part in the trial. At six months post-baseline, 154 participants (n=71 intervention group) had withdrawn from the study. At 12 months post-baseline, a further 45 participants had withdrawn, of which 22 participants were in the intervention group. Stated reasons for withdrawals included lack of time and/or interest.

**Lost to Follow Up**

Participants noted as ‘lost to follow up’ were those who were unable to be contacted due to moving worksite location, or who did not reply to email/phone contact by the research team. At six months post-baseline, 71 participants were lost to follow up (n=49 intervention group). Across the two groups, 69 participants were unable to be contacted and two participants had moved location. At 12 months post-baseline, a further 47 participants (n=30 intervention group) were lost to follow up, of which, five had retired or were off sick, 39 had moved location and a further three were unable to be contacted by the research team.

**Medium Term (12 mths)**

**Short-Term (6 mths)**

**Short-Term (0-6 mths)**

**Possible Mediators**

**Intervention**

**Long-Term (18 mths)**

Maintained behaviour change leads to maintained levels of:

Physical activity

Health

Mental wellbeing

Work absenteeism and presenteeism

Habit

Recovery self-efficacy

Social norms

Satisfaction with outcome expectancies

Further maintained behaviour change (> 3 yrs) leads to reduced risk of:

CVD

Cancer

Hypertension

Diabetes

Stroke

Multi-component intervention includes provision of points and rewards (non-cash financial incentives) contingent on meeting targeted behaviour goals

The following BCTs are included:

-immediate reward contingent on behaviour change; self-monitoring and feedback; info where/when to perform physical activity; specific goal setting; prompts and cues; action planning; barrier identification; social support; provision of info about health benefits of physical activity; habit formation; behavioural practice/rehearsal; behaviour substitution; adding objects to the environment; problem solving.

Gradual transition from focus on extrinsic motivation (rewards) to intrinsic motivation to increase physical activity by:

-reducing the extrinsic motivator by reducing the frequency of rewards given and rewards of lesser value;

-increase intrinsic motivators by increasing the emphasis on other BCTs in scheme, e.g. self-monitoring, feedback, goal setting, social support, planning, prompts and cues

Leads to increase in:

Physical activity

Health

Mental wellbeing

Work absenteeism and presenteeism

Recovery self-efficacy

Social norms

Perceptions of environment

Internet confidence

Outcome expectancy

Habit

Maintained behaviour change leads to:

-maintained increase of physical activity levels

**Uptake and Initiation:**

-self-efficacy

-intention

-outcome expectancies

-social norms

-discounting behaviour

-perceptions of workplace environment

-access to physical activity opportunities

-usage of PAL card

-usage of website

-web engagement and confidence

**Maintenance**

-habit

-recovery self-efficacy

-social norms

-satisfaction with outcome expectancies

**Underpinning Theoretical Framework**

Learning Theory

Social Cognitive Theory

**Figure 1.1. Logic model of the Physical Activity Loyalty scheme**

**
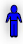

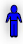

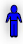

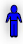
**

**Participant A**

*Timestamp 4: 05/01/2011; 13.25.02*

*Timestamp 2: 05/01/2011; 13.10.35*

*Timestamp 1 05/01/2011; 13.00.03*

**Sensor C**

**Sensor B**

**Sensor A**

**
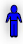
**

In the example shown in this diagram, participant A has walked for 25 minutes, and earned 25 points with a monetary value of £0.75.

**Figure 1.2. Diagram showing an example of how PAL scheme sensors recorded physical activity**

*Timestamp 3: 05/01/2011; 13.15.42*

**Participants receive personalised physical activity feedback**

**Participants incentivised to meet 150/mins physical activity/week**

**Figure 1.3. Diagram showing an overview of the components of the PAL scheme**


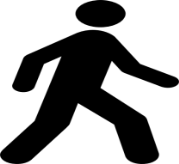

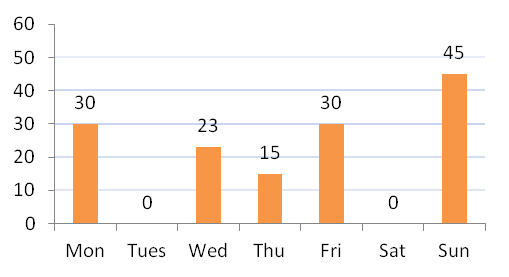


**Participants log-in to personal user account on website**


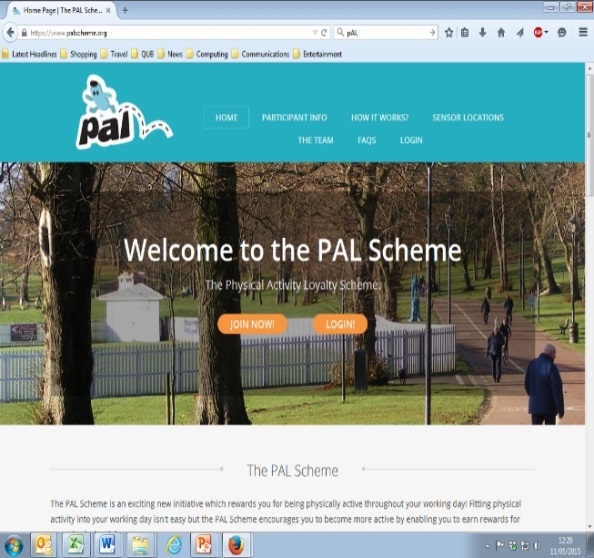


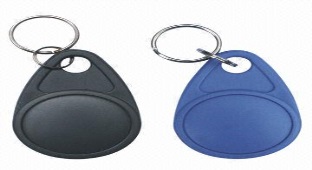

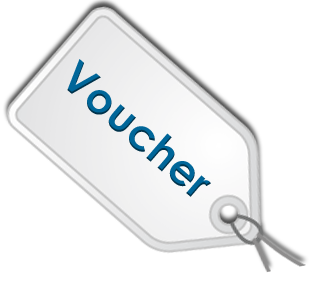


**Minutes of physical activity monitored by sensors and PAL fob keys**

**Physical activity points redeemable for retail vouchers**

Excluded (n=356)

- Not based at recruited worksite at least 4 h/day on at least 3 days/week (n=93)
- Current contract does not last the duration of the study (n=1)
- No longer wished to participate pre-randomisation (n=107)
- Did not provide baseline data (n=150)
- Unable to contact (n=5)

Assessed for eligibility (n=1,209)

**Enrolment**

Allocated to Control Group (n=396)

Valid pedometer data (n=359)

Allocated to Intervention Group (n=457)

Valid pedometer data (n=414)

Randomised (n=853)

**Allocation**

**Follow-up**

**six months**

Lost to follow-up (n=105)

- Withdrawn (n=83)
- Unable to contact (n=22)

Lost to follow-up (n=120)

- Withdrawn (n=71)
- Moved worksite (n=2)
- Unable to contact (n=47)

Analysed (n=236)

Excluded from analysis (n=55)

- Pedometer data not provided (n=38)
- Pedometer data not valid (n=17)

Analysed (n=249)

Excluded from analysis (n=88)

- Pedometer data not provided (n=63)
- Pedometer data not valid (n=25)

**Analysis**

**six months**

Lost to follow-up (n=40)

- Withdrawn (n=23)
- Moved worksite (n=16)
- Retired (n=1)

Lost to follow-up (n=52)

- Withdrawn (n=22)
- Moved worksite (n=23)
- Unable to contact (n=3)
- Retired/Sick (n=4)

**Follow-up**

**12 months**

Analysed (n=180)

Excluded from analysis (n=71)

- Pedometer data not provided (n=56)
- Pedometer data not valid (n=15)

**Analysis**

**12 months**

Analysed (n=210)

Excluded from analysis (n=75)

- Pedometer data not provided (n=59)
- Pedometer data not valid (n=16)

**Figure 1.4. CONSORT Flow diagram**

1. Black C, Frost D, Frost B. Health at work-an independent review of sickness absence [Internet]. 2011 [cited 2017 Feb 1]. Available from: https://www.gov.uk/government/publications/review-of-the-sickness-absence-system-in-great-britain. Archived at http://www.webcitation.org/73PZC0XcJ

2. Hamilton MT, Hamilton DG, Zderic TW. Role of low energy expenditure and sitting in obesity, metabolic syndrome, type 2 diabetes, and cardiovascular disease. Diabetes 2007 Nov 1;56(11):2655–2667. PMID:17827399

3. Hunter RF, Tully MA, Davis M, Stevenson M, Kee F. Physical activity loyalty cards for behavior change: a quasi-experimental study. Am J Prev Med 2013 Jul;45(1):56–63. PMID:23790989

4. Finkelstein EA, Haaland BA, Bilger M, Sahasranaman A, Sloan RA, Nang EEK, Evenson KR. Effectiveness of activity trackers with and without incentives to increase physical activity (TRIPPA): a randomised controlled trial. Lancet Diabetes Endocrinol 2016 Oct;219–229. [doi: 10.1016/S2213-8587(16)30284-4]

5. Giles EL, Robalino S, McColl E, Sniehotta FF, Adams J. The effectiveness of financial incentives for health behaviour change: systematic review and meta-analysis. Baradaran HR, editor. PLoS One United States; 2014 Mar 11;9(3):e90347. PMID:24618584

6. Craig P, Dieppe P, Macintyre S, Michie S, Nazareth I, Petticrew M. Developing and evaluating complex interventions: new guidance [Internet]. MRC. 2008 [cited 2014 Oct 22]. Available from: https://mrc.ukri.org/documents/pdf/complex-interventions-guidance/. Archived at http://www.webcitation.org/73O0aDvK8

7. Miller N, Dollard J. Social Learning and Imitation. New Haven, CT: Yale University Press; 1941. ISBN:10.2307/1169510

8. Carver CS, Scheier MF. Attention and Self-Regulation: a control-theory approach to human behaviour. New York, NY: Springer New York; 1981. ISBN:978-1-4612-5889-6

9. Bandura A. Self-Efficacy: The Exercise of Control. Worth Publishers; 1997. ISBN:978-0716728504

10. Marteau TM, Ashcroft RE, Oliver A. Using financial incentives to achieve healthy behaviour. BMJ 2009;338:b1415. PMID:19359291

11. Johnston M. What more can we learn from early learning theory? The contemporary relevance for behaviour change interventions. Br J Health Psychol 2016 Feb;21(1):1–10. [doi: 10.1111/bjhp.12165]

12. Deci E, Ryan R. Intrinsic motivation and Self-determination in human behaviour. 1985. ISBN:978-1-4899-2273-1
