# Supplementary material for: Predicting Outcomes from Engagement With Specific Components of an Internet-Based Physical Activity Intervention With Financial Incentives: Process Analysis of a Cluster Randomized Controlled Trial
Source: J Med Internet Res. 2019 Apr 19;21(4):e11394. doi: 10.2196/11394 (PMC6498305; doi:10.2196/11394)
Supplement: Multimedia Appendix 2 [file jmir_v21i4e11394_app2.docx]

**Multimedia Appendix 2. Description of assessed variables and baseline characteristics.**

**Table 2.1: Description of the assessed variables^1^**

| Concept | Items (n) | Example question item and answering option | α^2^ | Time-points | Reference |
| --- | --- | --- | --- | --- | --- |
| **Mediators** |  |  |  |  |  |
| Physical activity self-efficacy | 5 | How confident are you that you could do at least 150 minutes of physical activity over the next 7 days if each of the following situations arose? When I am tired. *Not at all confident* (1) to *Extremely confident* (5) | 0.86 | Baseline, 4 weeks | Marcus, Selby, Niaura, & Rossi, 1992 [1] |
| Intentions | 2 | I intend to do at least 150 minutes of physical activity over the next 7 days. *Strongly agree* (1) to *Strongly disagree* (7) | 0.92 | Baseline, 4 weeks | Fishbein & Ajzen, 1975 [2] |
| Outcome expectations | 8 | Doing at least 150 minutes of physical activity over the next 7 days will make me feel better physically. *Strongly agree* (1) to *Strongly disagree* (5) | 0.93 | Baseline, 4 weeks | Finch et al., 2005 [3] |
| Financial motivation | 7 | I am physically active because I want to earn extra money. *Not at all true for me* (1) to *Very true for me* (7) | 0.93 | Baseline, 4 weeks | Moller, Buscemi, McFadden, Hedeker, & Spring, 2014 [4] |
| Planning | 9 | I have made a detailed plan regarding when to do physical activity. *Completely disagree* (1) to *Totally agree* (4) | 0.96 | Baseline, 4 weeks, 6 months | Sniehotta et al., 2005 [5] |
| Social norms | 3 | Most people who are important to me do at least 150 minutes of physical activity over the next 7 days. *Completely true* (1) to *Completely false* (7) | 0.58 | Baseline, 4 weeks, 6 months | Ball et al., 2010 [6] |
| SDT: Identified regulation | 4 | It’s important for me to be physically active regularly. *Not at all true* (0) to *Very true for me* (4) | 0.84 | Baseline, 4 weeks, 6 months | Markland & Tobin, 2004 [7]; Wilson et al., 2007 [8] |
| SDT: Integrated regulation | 4 | I am physically active because it is consistent with my life goals. *Not at all true* (0) to *Very true for me* (4) | 0.93 | Baseline, 4 weeks, 6 months | Markland & Tobin, 2004 [7]; Wilson et al., 2007 [8] |
| SDT: Intrinsic motivation | 4 | I am physically active because it’s fun. *Not at all true* (0) to *Very true for me* (4) | 0.93 | Baseline, 4 weeks, 6 months | Markland & Tobin, 2004 [7]; Wilson et al., 2007 [8] |
| Habit | 4 | Doing at least 150 minutes of physical activity over the next 7 days is something that I do automatically. *Agree* (1) to *Disagree* (5) | 0.96 | Baseline, 6 months | Verplanken & Orbell, 2003 [9] |
| Workplace norms | 4 | I often see other work colleagues walking in my workplace. *Strongly agree* (1) to *Strongly disagree* (5) | 0.62 | Baseline, 6 months | Ball et al., 2010 [6] |
| Recovery self-efficacy | 4 | I am confident that I can return to doing at least 150 minutes of physical activity over the next 7 days again, even if I did not do 150 minutes of physical activity per week for some time because I felt weak. *Not at all true* (1) to *Exactly true* (4) | 0.81 | Baseline, 6 months | Scholz et al., 2005 [10] |
| Maintenance self-efficacy | 5 | I am confident that I can keep doing at least 150 minutes of physical activity over 7 days on a regular basis, even if I am troubled or worried. *Not at all true* (1) to *Exactly true* (4) | 0.95 | Baseline, 6 months | Scholz et al., 2005 [10] |
| Outcome satisfaction | 8 | How satisfied are you with any changes in how you feel physically, as a result of trying to do 150 minutes of physical activity per week? *Very dissatisfied* (-2) to *Very satisfied* (2) | 0.94 | Baseline, 6 months | Finch et al., 2005 [3]; Rothman, Baldwin, Hertel, & Fuglestad, 2011 [11] |
| **Moderators** |  |  |  |  |  |
| SF-8 Mental Component Score | 8 | During the past four weeks, how much have you been bothered by emotional problems (such as feeling anxious, depressed or irritable)? *Not at all* (1) to *Extremely* (5) | - | Baseline | Ware et al., 2001 [12] |
| SF-8 Physical Component Score | 8 | During the past four weeks, how much difficulty did you have doing your daily work, both at home and away from home, because of your physical health? *Not at all* (1) to *Could not do daily work* (5) | - | Baseline | Ware et al., 2001 [12] |
| EQ5D: Health State | 1 | How is your health today? *The worst health you can imagine* (0) to *The best health you can imagine* (100) | - | Baseline | EuroQol Group, 1991 [13] |
| EQ5D: Weighted Health Index | 5 | Please indicate which statements best describe your own health state today. Mobility: *I have no problems in walking about* (1) to *I am unable to walk about* (5) | - | Baseline | EuroQol Group, 1991 [13] |
| WEMWBS: Mental wellbeing scale | 14 | Over the last 2 weeks I’ve been feeling optimistic about the future. *None of the time* (1) to *All of the time* (5) | - | Baseline | Tennant et al., 2007 [14]; Lloyd & Devine, 2012 [15] |
| WE: Attractiveness | 4 | In my workplace environment it is pleasant to walk. *Strongly agree* (1) to *Strongly disagree* (5) | 0.67 | Baseline | Ogilvie et al., 2008 [16] |
| WE: Safety | 4 | In my workplace environment the roads are dangerous for cyclists. *Strongly agree* (1) to *Strongly disagree* (5) | 0.54 | Baseline | Ogilvie et al., 2008 [16] |
| WE: Accessibility | 3 | In my workplace environment there is convenient public transport. *Strongly agree* (1) to *Strongly disagree* (5) | 0.28 | Baseline | Ogilvie et al., 2008 [16] |
| WE: Availability | 3 | In my workplace environment there is a park within walking distance. *Strongly agree* (1) to *Strongly disagree* (5) | 0.27 | Baseline | Ogilvie et al., 2008 [16] |
| WE: Overall | 14 | *See examples above* | 0.74 | Baseline | Ogilvie et al., 2008 [16] |

EQ5D: EuroQol (five dimensions); SDT: Self-determination Theory; SF: short form; WE: Workplace environment; WEMWBS: Warwick-Edinburgh Mental Wellbeing Scale; WTA: willingness-to-accept.

^1^All questionnaire items were scored so that higher numerical values equated to higher levels of the construct. All self-report measures were collected online via Qualtrics (www.Qualtrics.com).

^2^Cronbach’s alpha calculated on baseline data.

**Table 2.2. Baseline characteristics of participants in the intervention group (n=457)**

|  | **Intervention group** |
| --- | --- |
| ***Characteristics of clusters*** | n = 19 |
| Number of participants; mean (range) | 24 (4 to 147) |
| Randomisation stratum, clusters (n, % participants) |  |
| Small (<20) | 11 (114, 25%) |
| Medium (20-50) | 5 (167, 37%) |
| Large (>50) | 1 (147, 32%) |
| Schools | 2 (29, 6%) |
| ***Characteristics of participants^a^*** | n=457 |
| Age (years) | 44.0 (9.3) |
| Female gender; n (%) | 329 (72%) |
| BMI (kg/m^2^) | 27.2 (5.6) |
| Income >£20k; n (%) | 341 (75%) |
| Education some higher level; n (%) | 295 (65%) |
| Marital status married/co-habiting; n (%) | 313 (68%) |
| Objective physical activity: pedometer steps (steps/day) | 7,977 (3,602) |
| Objective: physical activity category, n (%) |  |
| High (>7,500 steps/day) | 204 (45%) |
| Moderate (>2,500-≤7,500 steps/day) | 199 (44%) |
| Low (<2,500 steps/day) | 11 (2%) |
| GPAQ: minutes of work physical activity (minutes/week) | 42 (138) |
| GPAQ: minutes of MVPA (minutes/week) | 296 (342) |
| GPAQ: physical activity category, n (%) |  |
| High | 70 (15%) |
| Moderate | 140 (31%) |
| Low | 141 (31%) |
| SF-8: Mental Component Score | 48.0 (8.9) |
| SF-8: Physical Component Score | 52.5 (6.6) |
| EQ5D: Health State | 82.4 (13.8) |
| EQ5D: Weighted Health Index | 0.89 (0.11) |
| WEMWBS: Mental wellbeing scale | 50.2 (8.2) |
| Physical Activity Self-Efficacy scale | 2.91 (0.97) |
| HPQ: 4-week absolute absenteeism | 5.04 (41.3) |
| HPQ: Absolute presenteeism | 80.3 (13.6) |
| HPQ: Combined relative absenteeism and absolute presenteeism | 8.84 (12.57) |
| ***Environmental variables*** |  |
| WE: Attractiveness (4-20) | 10.83 (2.71) |
| WE: Safety (4-20) | 10.63 (2.47) |
| WE: Accessibility (3-15) | 9.27 (2.14) |
| WE: Availability (3-15) | 10.36 (2.00) |
|  |  |

**^a^Mean (SD) unless otherwise stated**

EQ5D: EuroQol; five dimensions; GPAQ: Global Physical Activity Questionnaire; HPQ: Health and Work Performance Questionnaire; MVPA: moderate- to vigorous-intensity physical activity; NHS: National Health Service; SD: standard deviation; SF: short form; WE: Workplace environment; WEMWBS: Warwick-Edinburgh Mental Wellbeing Scale.

1. Marcus BH, Selby VC, Niaura RS, Rossi JS. Self-efficacy and the stages of exercise behavior change. Res Q Exerc Sport 1992 Mar;63(1):60–66. PMID:1574662

2. Fishbein M, Ajzen I. Belief, attitude, intention and behavior: An introduction to theory and research. Reading,Addison-Wesley; 1975. ISBN:9780201020892

3. Finch EA, Linde JA, Jeffery RW, Rothman AJ, King CM, Levy RL. The effects of outcome expectations and satisfaction on weight loss and maintenance: correlational and experimental analyses--a randomized trial. Heal Psychol 2005 Nov;24(6):608–616. PMID:16287407

4. Moller AC, Buscemi J, McFadden HG, Hedeker D, Spring B. Financial motivation undermines potential enjoyment in an intensive diet and activity intervention. J Behav Med 2014 Oct;37(5):819–827. PMID:24142187

5. Sniehotta FF, Schwarzer R, Scholz U, Schüz B. Action planning and coping planning for long-term lifestyle change: theory and assessment. Eur J Soc Psychol John Wiley & Sons, Ltd.; 2005 Jul;35(4):565–576. [doi: 10.1002/ejsp.258]

6. Ball K, Jeffery RW, Abbott G, McNaughton SA, Crawford D. Is healthy behavior contagious: associations of social norms with physical activity and healthy eating. Int J Behav Nutr Phys Act BioMed Central; 2010 Jan 7;7(1):86. PMID:21138550

7. Markland D, Tobin V. A modification to the behavioural regulation in exercise questionnaire to include an assessment of amotivation. J Sport Exerc Psychol 2004;26(2):191–196. [doi: 10.1123/jsep.26.2.191]

8. Wilson PM, Rodgers WM, Loitz CC, Scime G. “It’s who I am … Really!’ The importance of integrated regulation in exercise contexts. J Appl Biobehav Res Wiley/Blackwell (10.1111); 2007 May 4;11(2):79–104. [doi: 10.1111/j.1751-9861.2006.tb00021.x]

9. Verplanken B, Orbell S. Reflections on past behavior: a self-report index of habit strength. J Appl Soc Psychol University of Bath; 2003 Oct 16;33(6):1313–1330. [doi: 10.1111/j.1559-1816.2003.tb01951.x]

10. Scholz U, Sniehotta FF, Schwarzer R. Predicting physical exercise in cardiac rehabilitation: the role of phase-specific self-efficacy beliefs. J Sport Exerc Psychol 2005;27(2):135–151. [doi: 10.5167/uzh-102309]

11. Rothman AJ, Baldwin AS, Hertel AW, Fuglestad PT. Self-regulation and behavior change: Disentangling behavioral initiation and behavioral maintenance. Handbook of self-regulation: Research, theory, and applications. Baumeister RF VK, editor. 2011. ISBN:9781462520459

12. Ware JE, Kosinski M, Dewey JE, Gandek B. How to score and interpret single-item health status measures: a manual for users of the of the SF-8 health survey. Lincoln RI: QualityMetric Incorporated; 2001. ISBN:9781891810084

13. EuroQol Group. EuroQol: a new facility for the measurement of health-related quality of life. Health Policy (New York) 1991;16:199–208. PMID:10109801

14. Tennant R, Hiller L, Fishwick R, Platt S, Joseph S, Weich S, Parkinson J, Secker J, Stewart-Brown S. The Warwick-Edinburgh Mental Well-being Scale (WEMWBS): development and UK validation. Health Qual Life Outcomes BioMed Central; 2007;5(1):63. PMID:18042300

15. Lloyd K, Devine P. Psychometric properties of the Warwick–Edinburgh Mental Well-being Scale (WEMWBS) in Northern Ireland. J Ment Heal 2012 Jun 10;21(3):257–263. PMID:22574955

16. Ogilvie D, Mitchell R, Mutrie N, Petticrew M, Platt S. Perceived characteristics of the environment associated with active travel: development and testing of a new scale. Int J Behav Nutr Phys Act BioMed Central; 2008 Jan 30;5(1):32. PMID:18513430
