# Supplementary material for: Predicting Outcomes from Engagement With Specific Components of an Internet-Based Physical Activity Intervention With Financial Incentives: Process Analysis of a Cluster Randomized Controlled Trial
Source: J Med Internet Res. 2019 Apr 19;21(4):e11394. doi: 10.2196/11394 (PMC6498305; doi:10.2196/11394)
Supplement: Multimedia Appendix 3 [file jmir_v21i4e11394_app3.docx]

**Multimedia Appendix 3. Results of univariable random-effects regressions with individual mediators as dependent variables and engagement indicators as independent variables.**

**Table 3.1. Results of univariable random-effects regressions with individual mediators as dependent variables and engagement in intervention components as independent variables among intervention group participants providing six month data**

|  | % intervention days participants walked for at least 10 min captured via the physical activity monitoring system ^a^ | | | % intervention weeks participants logged onto the website^b^ | | | % earned points redeemed^c^ | | |
| --- | --- | --- | --- | --- | --- | --- | --- | --- | --- |
| OUTCOME | n | *b* (SE) | *P* value | n | *b* (SE) | *P* value | n | *b* (SE) | *P* value |
|  |  |  |  |  |  |  |  |  |  |
| Planning | 228 | 0.003 (0.002) | .18 | 229 | 0.003 (0.002) | .053 | 228 | 0.001 (0.001) | .56 |
| Social norms | 230 | 0.004 (0.003) | .19 | 230 | 0.002 (0.003) | .36 | 230 | 0.003 (0.001) | **.01** |
| Identified regulation | 241 | 0.002 (0.002) | .33 | 243 | 0.002 (0.001) | .06 | 241 | 0.000 (0.001) | .87 |
| Integrated regulation | 238 | 0.007 (0.002) | **.004** | 240 | 0.004 (0.002) | **.02** | 238 | 0.000 (0.001) | .82 |
| Intrinsic motivation | 238 | 0.003 (0.001) | **.04** | 240 | 0.002 (0.001) | **.04** | 238 | 0.000 (0.001) | .72 |
| Habit | 233 | 0.007 (0.003) | **.01** | 235 | 0.004 (0.002) | **.03** | 233 | 0.000 (0.002) | .98 |
| Workplace norms | 239 | 0.000 (0.002) | .83 | 241 | 0.001 (0.002) | .55 | 239 | 0.001 (0.001) | .45 |
| Recovery self-efficacy | 241 | 0.004 (0.003) | .15 | 243 | 0.002 (0.001) | .18 | 241 | 0.000 (0.001) | .81 |
| Maintenance self-efficacy | 242 | 0.002 (0.003) | .55 | 244 | 0.002 (0.001) | .27 | 242 | -0.001 (0.001) | .36 |
| Outcome satisfaction | 225 | 0.005 (0.002) | **.01** | 225 | 0.004 (0.001) | **.01** | 225 | 0.001 (0.001) | .11 |
|  |  |  |  |  |  |  |  |  |  |

^a^Percentage of days participants were recorded walking for at least 10 min as captured via the physical activity monitoring system.

^b^Percentage of weeks participants logged onto the website at least once.

^c^Percentage of total accumulated points which the participant had redeemed by six months.

NB. Results are adjusted for randomisation stratum, season, baseline pedometer steps/day and baseline mediators with cluster-adjusted standard errors and *P* values.

**Table 3.2. Results of univariable random-effects regressions with individual mediators as dependent variables and frequency of hits on each section of the website as independent variables among intervention group participants providing six month data**

|  |  | Monitoring and feedback^a^ | | | Rewards^a^ | | Maps^a^ | | Health information: Physical activity^a^ | | | Health information: Other^a^ | | | Discussion forums^a^ | | | Number of sections^b^ | | |
| --- | --- | --- | --- | --- | --- | --- | --- | --- | --- | --- | --- | --- | --- | --- | --- | --- | --- | --- | --- | --- |
| OUTCOME | n | | *b* (SE) | *P* value | *b* (SE) | *P* value | *b* (SE) | *P* value | *b* (SE) | *P* value | *b* (SE) | | *P* value | *b* (SE) | | *P* value | *b* (SE) | | *P* value |  |
|  |  | |  |  |  |  |  |  |  |  |  | |  |  | |  |  | |  |  |
| Planning | 229 | | -0.04 (0.01) | **<.001** | 0.01 (0.01) | .59 | -0.01 (0.01) | .55 | -0.05 (0.05) | .36 | -0.05 (0.03) | | .06 | 0.00 (0.01) | | .80 | 0.03 (0.04) | | .43 |  |
| Social norms | 230 | | 0.01 (0.02) | .61 | -0.02 (0.02) | .31 | -0.03 (0.02) | .09 | -0.05 (0.06) | .46 | 0.01 (0.03) | | .68 | -0.03 (0.01) | | **.03** | 0.04 (0.04) | | .29 |  |
| Identified regulation | 243 | | 0.02 (0.01) | **.04** | -0.01 (0.01) | .61 | 0.01 (0.01) | .52 | 0.06 (0.04) | .08 | 0.04 (0.04) | | .33 | 0.01 (0.01) | | .15 | 0.07 (0.02) | | **<.001** |  |
| Integrated regulation | 240 | | 0.03 (0.01) | **.02** | 0.00 (0.01) | .97 | -0.02 (0.01) | .10 | 0.11 (0.05) | **.03** | 0.06 (0.06) | | .27 | -0.02 (0.01) | | **.03** | 0.09 (0.03) | | **.005** |  |
| Intrinsic motivation | 240 | | 0.01 (0.01) | .29 | -0.01 (0.01) | .67 | 0.00 (0.01) | .88 | 0.06 (0.05) | .23 | 0.05 (0.05) | | .34 | -0.00 (0.01) | | .61 | 0.04 (0.03) | | .12 |  |
| Habit | 235 | | 0.00 (0.02) | .99 | -0.01 (0.02) | .79 | 0.01 (0.02) | .50 | 0.06 (0.06) | .36 | -0.06 (0.07) | | .40 | -0.02 (0.01) | | .07 | 0.01 (0.04) | | .73 |  |
| Workplace norms | 241 | | 0.01 (0.01) | .70 | -0.00 (0.01) | .96 | 0.00 (0.01) | .95 | 0.06 (0.04) | .18 | 0.01 (0.04) | | .71 | 0.00 (0.01) | | .72 | 0.03 (0.03) | | .28 |  |
| Recovery self-efficacy | 243 | | -0.01 (0.01) | .46 | -0.01 (0.01) | .68 | -0.01 (0.01) | .11 | 0.05 (0.04) | .24 | 0.03 (0.03) | | .30 | 0.00 (0.01) | | .80 | 0.03 (0.02) | | .10 |  |
| Maintenance self-efficacy | 244 | | -0.01 (0.01) | .53 | -0.01 (0.01) | .40 | -0.02 (0.02) | .31 | -0.03 (0.07) | .65 | -0.02 (0.04) | | .59 | -0.01 (0.01) | | .06 | 0.00 (0.04) | | .94 |  |
| Outcome satisfaction | 225 | | -0.01 (0.01) | .38 | 0.01 (0.01) | .57 | -0.01 (0.01) | .44 | -0.04 (0.04) | .31 | -0.02 (0.02) | | .35 | 0.01 (0.01) | | .11 | 0.05 (0.04) | | .16 |  |
|  |  | |  |  |  |  |  |  |  |  |  | |  |  | |  |  | |  |  |

^a^Frequency of hits (i.e. total number of hits for every ten days the participant accessed the website).

^b^Number of sections accessed on website at least once (range 0-6).

NB. Results are adjusted for randomisation stratum, season, baseline pedometer steps/day and baseline mediators with cluster-adjusted standard errors and *P* values.
