# Supplementary material for: Predicting Outcomes from Engagement With Specific Components of an Internet-Based Physical Activity Intervention With Financial Incentives: Process Analysis of a Cluster Randomized Controlled Trial
Source: J Med Internet Res. 2019 Apr 19;21(4):e11394. doi: 10.2196/11394 (PMC6498305; doi:10.2196/11394)
Supplement: Multimedia Appendix 4 [file jmir_v21i4e11394_app4.docx]

**Multimedia Appendix 4. Univariable and multivariable Cox regression analyses.**

#### Table 4.1. Univariable and multivariable Cox regression showing association of non-usage attrition risk for recording daily activity via the physical activity monitoring system with baseline socio-demographic, psychosocial and environmental variables, and physical activity

|  | Univariable analysis | | | Multivariable analysis^a^ | | |
| --- | --- | --- | --- | --- | --- | --- |
| OUTCOME | n | Hazard ratio (95% CI) | *P* value | n | Hazard ratio (95% CI) | *P* value |
|  |  |  |  |  |  |  |
| ***Socio-demographic variables*** |  |  |  |  |  |  |
| Age (decades) | 421 | 0.94 (0.84, 1.06) | .32 |  |  |  |
| Gender (Female *vs* Male) | 422 | 0.99 (0.69, 1.40) | .94 |  |  |  |
| BMI (kg/m^2^) | 406 | 1.00 (0.98, 1.02) | .79 |  |  |  |
| Income (>£20k *vs* ≤£20k) | 411 | 1.11 (0.78, 1.59) | .55 |  |  |  |
| Some higher education (Yes *vs* No) | 412 | 0.94 (0.57, 1.55) | .81 |  |  |  |
| Married/co-habiting (Yes *vs* No) | 412 | 1.30 (0.95, 1.79) | .10 |  |  |  |
| SF-8: Mental Component Score | 415 | 0.99 (0.98, 1.00) | **.03** |  |  |  |
| SF-8: Physical Component Score | 415 | 0.99 (0.98, 1.00) | .08 |  |  |  |
| EQ5D: Health State | 367 | 1.00 (0.99, 1.00) | .68 |  |  |  |
| EQ5D: Weighted Health Index | 368 | 0.50 (0.19, 1.32) | .16 |  |  |  |
| WEMWBS: Mental wellbeing scale | 413 | 1.00 (0.99, 1.01) | .43 |  |  |  |
| ***Mediator variables*** |  |  |  |  |  |  |
| Physical activity self-efficacy | 410 | 0.94 (0.83, 1.07) | .34 |  |  |  |
| Intentions | 405 | 1.00 (0.95, 1.06) | .93 |  |  |  |
| Outcome expectations | 389 | 1.05 (0.83, 1.32) | .70 |  |  |  |
| Financial motivation | 409 | 0.99 (0.91, 1.07) | .77 |  |  |  |
| Planning | 386 | 0.96 (0.82, 1.11) | .56 |  |  |  |
| Social norms | 389 | 0.94 (0.86, 1.04) | .24 |  |  |  |
| Identified regulation | 409 | 0.87 (0.79, 0.96) | **.005** | 407 | 0.88 (0.81, 0.97) | **.009** |
| Integrated regulation | 409 | 0.93 (0.85, 1.02) | .14 |  |  |  |
| Intrinsic motivation | 408 | 0.91 (0.83, 0.99) | **.04** |  |  |  |
| Habit | 407 | 0.96 (0.89, 1.04) | .32 |  |  |  |
| Workplace norms | 409 | 1.05 (0.92, 1.19) | .47 |  |  |  |
| Recovery self-efficacy | 408 | 0.86 (0.77, 0.96) | **.005** | 407 | 0.88 (0.80, 0.98) | **.02** |
| Maintenance self-efficacy | 408 | 0.96 (0.83, 1.10) | .55 |  |  |  |
| Outcome satisfaction | 377 | 0.95 (0.80, 1.13) | .57 |  |  |  |
| ***Environmental variables*** |  |  |  |  |  |  |
| WE: Attractiveness | 409 | 1.06 (0.98, 1.14) | .17 |  |  |  |
| WE: Safety | 410 | 1.06 (1.01, 1.11) | **.02** | 407 | 1.07 (1.02, 1.11) | **.008** |
| WE: Accessibility | 410 | 1.00 (0.95, 1.05) | .99 |  |  |  |
| WE: Availability | 410 | 0.97 (0.93, 1.01) | .11 |  |  |  |
| ***Physical activity*** |  |  |  |  |  |  |
| Pedometer steps/day | 388 | 1.00 (1.00, 1.00) | .15 |  |  |  |
|  |  |  |  |  |  |  |

NB. Time variable = number of days until first two week lapse from recording daily activity via physical activity monitoring system; Event variable = 1 (non-usage attrition occurred) or 0 (non-usage attrition did not occur).

^a^Univariable analyses were conducted on all predictor variables and those with *P*<.05 were included in a multivariable model with backwards elimination of the predictor with the highest *P* value until all included predictors had *P*<.05.

#### Table 4.2. Univariable and multivariable Cox regression showing association of website non-usage attrition risk with baseline socio-demographics, psychosocial and environmental variables, and physical activity

|  | Univariable analysis | | | Multivariable analysis^a^ | | |
| --- | --- | --- | --- | --- | --- | --- |
| OUTCOME | n | Hazard ratio (95% CI) | *P* value | n | Hazard ratio  (95% CI) | *P* value |
|  |  |  |  |  |  |  |
| ***Socio-demographics*** |  |  |  |  |  |  |
| Age (decades) | 417 | 0.95 (0.87, 1.03) | .18 |  |  |  |
| Gender (Female *vs* Male) | 418 | 1.01 (0.84, 1.20) | .94 |  |  |  |
| BMI (kg/m^2^) | 403 | 0.99 (0.98, 1.01) | .29 |  |  |  |
| Income (>£20k *vs* ≤£20k) | 407 | 1.17 (0.95, 1.43) | .14 |  |  |  |
| Some higher education (Yes *vs* No) | 408 | 0.82 (0.62, 1.09) | .18 |  |  |  |
| Married/co-habiting (Yes *vs* No) | 408 | 1.22 (0.94, 1.58) | .14 |  |  |  |
| SF-8: Mental Component Score | 411 | 1.00 (0.99, 1.01) | .42 |  |  |  |
| SF-8: Physical Component Score | 411 | 1.00 (0.99, 1.02) | .83 |  |  |  |
| EQ5D: Health State | 366 | 1.00 (0.99, 1.00) | .29 |  |  |  |
| EQ5D: Weighted Health Index | 367 | 0.34 (0.13, 0.87) | **.02** | 366 | 0.33 (0.12, 0.91) | **.03** |
| WEMWBS: Mental wellbeing scale | 409 | 1.00 (0.99, 1.02) | .84 |  |  |  |
| ***Mediator variables*** |  |  |  |  |  |  |
| Physical activity self-efficacy | 406 | 0.95 (0.88, 1.03) | .24 |  |  |  |
| Intentions | 401 | 1.03 (0.96, 1.09) | .44 |  |  |  |
| Outcome expectations | 388 | 1.10 (0.93, 1.32) | .27 |  |  |  |
| Financial motivation | 405 | 0.94 (0.88, 0.99) | **.02** | 366 | 0.93 (0.87, 0.99) | **.02** |
| Planning | 382 | 0.92 (0.80, 1.06) | .26 |  |  |  |
| Social norms | 384 | 1.04 (0.93, 1.17) | .47 |  |  |  |
| Identified regulation | 405 | 1.01 (0.88, 1.16) | .88 |  |  |  |
| Integrated regulation | 405 | 1.02 (0.92, 1.12) | .75 |  |  |  |
| Intrinsic motivation | 404 | 1.02 (0.91, 1.14) | .74 |  |  |  |
| Habit | 403 | 0.99 (0.92, 1.07) | .78 |  |  |  |
| Workplace norms | 405 | 1.07 (0.95, 1.21) | .26 |  |  |  |
| Recovery self-efficacy | 404 | 0.93 (0.81, 1.07) | .32 |  |  |  |
| Maintenance self-efficacy | 404 | 1.07 (0.94, 1.22) | .29 |  |  |  |
| Outcome satisfaction | 372 | 0.93 (0.78, 1.12) | .46 |  |  |  |
| ***Environmental variables*** |  |  |  |  |  |  |
| WE: Attractiveness | 405 | 1.04 (0.99, 1.10) | .12 |  |  |  |
| WE: Safety | 405 | 1.04 (0.99, 1.10) | .09 |  |  |  |
| WE: Accessibility | 406 | 0.99 (0.96, 1.01) | .32 |  |  |  |
| WE: Availability | 406 | 0.96 (0.93, 0.99) | **.003** | 366 | 0.96 (0.93, 0.99) | **.02** |
| ***Physical activity*** |  |  |  |  |  |  |
| Pedometer steps/day | 383 | 1.00 (1.00, 1.00) | .09 |  |  |  |
|  |  |  |  |  |  |  |

NB. Time variable = number of days until first two week lapse from logging onto the website; Event variable = 1 (non-usage attrition occurred) or 0 (non-usage attrition did not occur).

^a^Univariable analyses were conducted on all predictor variables and those with *P*<.05 were included in a multivariable model with backwards elimination of the predictor with the highest *P* value until all included predictors had *P*<.05.
