# Supplementary material for: Predicting Outcomes from Engagement With Specific Components of an Internet-Based Physical Activity Intervention With Financial Incentives: Process Analysis of a Cluster Randomized Controlled Trial
Source: J Med Internet Res. 2019 Apr 19;21(4):e11394. doi: 10.2196/11394 (PMC6498305; doi:10.2196/11394)
Supplement: Multimedia Appendix 5 [file jmir_v21i4e11394_app5.docx]

**Multimedia Appendix 5. Univariable and multivariable Cox regression analyses defining nonusage attrition as occurring at the first lapse from use of 1 month or longer**

Defining non-usage attrition as occurring at the first lapse from use of one month (30 days) or longer, the median usage (i.e. the time by which 50% of participants’ usage had lapsed) was 121 days for use of the physical activity monitoring system to record daily activity (n=211/422) and 46 days for use of the website (n=209/418). Non-usage attrition of the physical activity monitoring system to record daily activity occurred for 55% of participants (n=232/422), and website non-usage attrition occurred for 82% of participants (n=344/418).

Univariable and multivariable Cox regression analyses are presented in tables 6.1 and 6.2 below. The multivariable analysis for use of the physical activity monitoring system to record daily activity showed that having higher levels of identified regulation at baseline (hazard ratio [HR]=0.77, 95% CI: 0.66, 0.89) reduced the risk of attrition. In contrast, having a higher perception of the safety of the workplace environment for physical activity at baseline (HR=1.09, 95% CI: 1.02, 1.17) was associated with a higher risk of attrition. The multivariable analysis for website use showed that being older (HR=0.93, 95% CI: 0.87, 1.00), or having higher levels of physical activity self-efficacy at baseline (HR=0.89, 95% CI: 0.79, 0.99) reduced the risk of attrition. In contrast, having a higher perception of the safety of the workplace environment for physical activity at baseline (HR=1.07, 95% CI: 1.02, 1.12) was associated with a higher risk of attrition. Formal tests and visual inspection of plots showed no evidence for violation of the proportional-hazards assumption for the multivariable models.

#### Table 5.1. Univariable and multivariable Cox regression showing association of non-usage attrition risk for recording daily activity via the physical activity monitoring system with baseline socio-demographic, psychosocial and environmental variables, and physical activity

|  | Univariable analysis | | | Multivariable analysis^a^ | | |
| --- | --- | --- | --- | --- | --- | --- |
| OUTCOME | n | Hazard ratio (95% CI) | *P* value | n | Hazard ratio (95% CI) | *P* value |
|  |  |  |  |  |  |  |
| ***Socio-demographic variables*** |  |  |  |  |  |  |
| Age (decades) | 421 | 0.93 (0.83, 1.04) | .21 |  |  |  |
| Gender (Female *vs* Male) | 422 | 0.92 (0.55, 1.54) | .75 |  |  |  |
| BMI (kg/m^2^) | 406 | 1.01 (0.98, 1.03) | .63 |  |  |  |
| Income (>£20k *vs* ≤£20k) | 411 | 1.22 (0.82, 1.81) | .32 |  |  |  |
| Some higher education (Yes *vs* No) | 412 | 1.03 (0.59, 1.81) | .91 |  |  |  |
| Married/co-habiting (Yes *vs* No) | 412 | 0.82 (0.59, 1.15) | .25 |  |  |  |
| SF-8: Mental Component Score | 415 | 0.99 (0.98, 1.00) | .08 |  |  |  |
| SF-8: Physical Component Score | 415 | 0.99 (0.98, 1.01) | .40 |  |  |  |
| EQ5D: Health State | 367 | 1.00 (0.99, 1.01) | .82 |  |  |  |
| EQ5D: Weighted Health Index | 368 | 1.05 (0.42, 2.60) | .92 |  |  |  |
| WEMWBS: Mental wellbeing scale | 413 | 0.99 (0.97, 1.00) | .06 |  |  |  |
| ***Mediator variables*** |  |  |  |  |  |  |
| Physical activity self-efficacy | 410 | 0.87 (0.74, 1.02) | .09 |  |  |  |
| Intentions | 405 | 1.03 (0.99, 1.08) | .12 |  |  |  |
| Outcome expectations | 389 | 1.00 (0.79, 1.26) | .97 |  |  |  |
| Financial motivation | 409 | 0.96 (0.87, 1.06) | .42 |  |  |  |
| Planning | 386 | 0.89 (0.76, 1.03) | .12 |  |  |  |
| Social norms | 389 | 0.94 (0.88, 1.00) | **.04** |  |  |  |
| Identified regulation | 409 | 0.75 (0.68, 0.91) | **.001** | 409 | 0.77 (0.66, 0.89) | **<.001** |
| Integrated regulation | 409 | 0.85 (0.74, 0.97) | **.02** |  |  |  |
| Intrinsic motivation | 408 | 0.87 (0.74, 1.01) | .06 |  |  |  |
| Habit | 407 | 0.93 (0.85, 1.03) | .15 |  |  |  |
| Workplace norms | 409 | 1.15 (0.99, 1.33) | .07 |  |  |  |
| Recovery self-efficacy | 408 | 0.86 (0.72, 1.03) | .11 |  |  |  |
| Maintenance self-efficacy | 408 | 0.88 (0.79, 0.98) | **.02** |  |  |  |
| Outcome satisfaction | 377 | 1.03 (0.86, 1.24) | .74 |  |  |  |
| ***Environmental variables*** |  |  |  |  |  |  |
| WE: Attractiveness | 409 | 1.09 (0.99, 1.19) | .08 |  |  |  |
| WE: Safety | 410 | 1.08 (1.01, 1.16) | **.03** | 409 | 1.09 (1.02, 1.17) | **.01** |
| WE: Accessibility | 410 | 1.04 (0.97, 1.13) | .29 |  |  |  |
| WE: Availability | 410 | 0.96 (0.91, 1.00) | .06 |  |  |  |
| ***Physical activity*** |  |  |  |  |  |  |
| Pedometer steps/day | 388 | 1.00 (1.00, 1.00) | .15 |  |  |  |
|  |  |  |  |  |  |  |

NB. Time variable = number of days until first one month (30 day) lapse from recording daily activity via physical activity monitoring system; Event variable = 1 (non-usage attrition occurred) or 0 (non-usage attrition did not occur).

^a^Univariable analyses were conducted on all predictor variables and those with *P*<.05 were included in a multivariable model with backwards elimination of the predictor with the highest *P* value until all included predictors had *P*<.05.

#### Table 5.2. Univariable and multivariable Cox regression showing association of website non-usage attrition risk with baseline socio-demographics, psychosocial and environmental variables, and physical activity

|  | Univariable analysis | | | Multivariable analysis^a^ | | |
| --- | --- | --- | --- | --- | --- | --- |
| OUTCOME | n | Hazard ratio (95% CI) | *P* value | n | Hazard ratio  (95% CI) | *P* value |
|  |  |  |  |  |  |  |
| ***Socio-demographics*** |  |  |  |  |  |  |
| Age (decades) | 417 | 0.92 (0.86, 0.99) | **.02** | 404 | 0.93 (0.87, 1.00) | **.04** |
| Gender (Female *vs* Male) | 418 | 1.06 (0.83, 1.34) | .65 |  |  |  |
| BMI (kg/m^2^) | 403 | 0.98 (0.97, 1.00) | .08 |  |  |  |
| Income (>£20k *vs* ≤£20k) | 407 | 1.22 (0.86, 1.73) | .27 |  |  |  |
| Some higher education (Yes *vs* No) | 408 | 0.88 (0.59, 1.31) | .53 |  |  |  |
| Married/co-habiting (Yes *vs* No) | 408 | 0.92 (0.70, 1.21) | .55 |  |  |  |
| SF-8: Mental Component Score | 411 | 0.99 (0.98, 1.00) | .09 |  |  |  |
| SF-8: Physical Component Score | 411 | 1.01 (1.00, 1.03) | .15 |  |  |  |
| EQ5D: Health State | 366 | 1.00 (1.00, 1.01) | .52 |  |  |  |
| EQ5D: Weighted Health Index | 367 | 1.23 (0.45, 3.33) | .69 |  |  |  |
| WEMWBS: Mental wellbeing scale | 409 | 1.00 (0.99, 1.01) | .67 |  |  |  |
| ***Mediator variables*** |  |  |  |  |  |  |
| Physical activity self-efficacy | 406 | 0.91 (0.82, 1.00) | **.04** | 404 | 0.89 (0.79, 0.99) | **.04** |
| Intentions | 401 | 1.03 (0.98, 1.08) | .24 |  |  |  |
| Outcome expectations | 388 | 1.05 (0.80, 1.37) | .74 |  |  |  |
| Financial motivation | 405 | 0.96 (0.89, 1.03) | .24 |  |  |  |
| Planning | 382 | 0.83 (0.71, 0.98) | **.02** |  |  |  |
| Social norms | 384 | 0.95 (0.88, 1.02) | .13 |  |  |  |
| Identified regulation | 405 | 0.94 (0.81, 1.08) | .38 |  |  |  |
| Integrated regulation | 405 | 0.95 (0.83, 1.08) | .43 |  |  |  |
| Intrinsic motivation | 404 | 0.96 (0.86, 1.06) | .43 |  |  |  |
| Habit | 403 | 0.98 (0.95, 1.01) | .26 |  |  |  |
| Workplace norms | 405 | 0.99 (0.85, 1.16) | .92 |  |  |  |
| Recovery self-efficacy | 404 | 0.88 (0.75, 1.03) | .11 |  |  |  |
| Maintenance self-efficacy | 404 | 0.92 (0.82, 1.04) | .20 |  |  |  |
| Outcome satisfaction | 372 | 0.89 (0.71, 1.12) | .32 |  |  |  |
| ***Environmental variables*** |  |  |  |  |  |  |
| WE: Attractiveness | 405 | 1.05 (0.98, 1.12) | .16 |  |  |  |
| WE: Safety | 405 | 1.06 (1.01, 1.11) | **.01** | 404 | 1.07 (1.02, 1.12) | **.005** |
| WE: Accessibility | 406 | 1.00 (0.95, 1.06) | .98 |  |  |  |
| WE: Availability | 406 | 0.97 (0.93, 1.02) | .28 |  |  |  |
| ***Physical activity*** |  |  |  |  |  |  |
| Pedometer steps/day | 383 | 1.00 (1.00, 1.00) | .42 |  |  |  |
|  |  |  |  |  |  |  |

NB. Time variable = number of days until first one month (30 day) lapse from logging onto the website; Event variable = 1 (non-usage attrition occurred) or 0 (non-usage attrition did not occur).

^a^Univariable analyses were conducted on all predictor variables and those with *P*<.05 were included in a multivariable model with backwards elimination of the predictor with the highest *P* value until all included predictors had *P*<.05.
